# Effects of Hydrophobic Modifications on the Solution Self-Assembly of P(DMAEMA-co-QDMAEMA)-*b*-POEGMA Random Diblock Copolymers

**DOI:** 10.3390/polym13030338

**Published:** 2021-01-21

**Authors:** Martha Kafetzi, Stergios Pispas

**Affiliations:** Theoretical and Physical Chemistry Institute, National Hellenic Research Foundation, 48 Vassileos Constantinou Avenue, 11635 Athens, Greece; mkafetzi91@gmail.com

**Keywords:** amphiphilic block copolymers, random blocks, RAFT polymerization, quaternization, hydrophobic modification, solution properties, pH-response, temperature-induced response, encapsulation of indomethacin

## Abstract

In this work, the synthesis and the aqueous solution self-assembly behavior of novel partially hydrophobically modified poly(2-(dimethylamino) ethyl methacrylate)-b-poly(oligo(ethylelene glycol) methyl ether methacrylatetabel) pH and temperature responsive random diblock copolymers (P(DMAEMA-co-Q_6/12_DMAEMA)-*b*-POEGMA), are reported. The chemical modifications were accomplished via quaternization with 1-iodohexane (Q_6_) and 1-iodododecane (Q_12_) and confirmed by ^1^H-NMR spectroscopy. The successful synthesis of PDMAEMA-*b*-POEGMA precursor block copolymers was conducted by RAFT polymerization. The partial chemical modification of the diblocks resulted in the permanent attachment of long alkyl chains on the amine groups of the PDMAEMA block and the presence of tertiary and quaternary amines randomly distributed within the PDMAEMA block. Light scattering techniques confirmed that the increased hydrophobic character results in the formation of nanoaggregates of high mass and tunable pH and temperature response. The characteristics of the aggregates are also affected by the aqueous solution preparation protocol, the nature of the quaternizing agent and the quaternization degree. The incorporation of long alkyl chains allowed the encapsulation of indomethacin within the amphiphilic diblock copolymer aggregates. Nanostructures of increased size were detected due to the encapsulation of indomethacin into the interior of the hydrophobic domains. Drug release studies demonstrated that almost 50% of the encapsulated drug can be released on demand by aid of ultrasonication.

## 1. Introduction

Amphiphilic block copolymers (AmBCs) have gained extensive scientific interest over the last decades in studies regarding the synthesis of novel materials, by employing facile polymerization techniques, along with the at-length apperception of their behavioral features allowing for their utilization in a broad field of applications [1,2]. The most fascinating feature of AmBCs is the formation of nanosized structures via self-assembly processes when inserted in aqueous media and thus their potential implementation in nanotechnology based concepts [3,4,5]. This impressive ability along with other capacities, such as the structural adjustment to surrounding media, the response to physicochemical stimuli and the interaction with biomolecules, ensures the eligibility of AmBCs for advanced biomedical applications, such as gene and drug delivery, and theranostics [6,7]. According to each application requirement, control over the structural and morphological features of the nanoparticulate structures may be imprinted during the synthesis process [3]. Molecular weight, composition and architecture are parameters of major importance for achieving nanostructures of specific morphology and function [3]. Random copolymers are an emerging class of synthetic polymeric materials that regained the interest of the scientific community only in recent years [8]. Progress in controlled radical polymerization methodologies brought the synthesis and investigation of the random copolymers to the forefront again as their molecular characteristics can be regulated in a considerable degree, taking also into account that random copolymers excel block copolymer in certain features [9,10]. Briefly, the incorporation of more than one functional monomeric unit can occur at one synthesis step and the supramolecular structures formed depend upon the molecular weight, the complexity of the system (number and nature of comonomers) and the functionality of the chosen comonomers [8,9,10,11]. Generally, random copolymers when inserted in aqueous media may self-fold intramolecularly into unimolecular micelle-like structures [9,10,11]. On the other hand, block copolymers more often self-assemble into core-shell type multichain micelles [12]. The hydrophobic/hydrophilic fraction of amphiphilic random copolymers plays the most significant role on their self-organization behavior. Considerably lower hydrophobic content results in intramolecular self-folding and formation of single chain nanoparticles with hydrophobic core domains consisted of the hydrophobic units [10,11,13,14]. When the hydrophobic content increases, intermolecular self-assembly occurs and multichain aggregates are formed [13,14,15,16].

Intelligent or stimuli-responsive polymers are an essential class of synthetic polymers that differ from conventional polymers because they are reactive to microenvironmental changes and as a result, they transit into a different physicochemical state, forming supramolecular nanostructures of diverse morphologies [2,17,18,19,20]. The interchange between hydrated and dehydrated state appoints them as interesting nanocarriers for gene and drug delivery applications [18,21,22,23,24,25,26]. These structural, physical or chemical alterations may be reversible or irreversible [27]. The introduction of stimuli-responsive polymeric units in a polymeric system enhances the system’s functionality and structural intricacy at the molecular and supramolecular level [28]. The capability of amphiphilic stimuli-sensitive random and/or block copolymers to self-assemble can be handled after being subjected to one or more external stimuli such as temperature, pH and ionic strength [25,29,30,31,32,33]. Polymers exhibiting pH and temperature sensitivity are two classes of intriguing intelligent polymers due to their utilization in bioapplications [18]. Poly(2-(dimethylamino)ethyl methacrylate) (PDMAEMA) is a pH and temperature responsive polymer [34]. The transition to a more hydrophobic state from a hydrophilic one occurs at a lower critical solution temperature (LCST) of about 40–50 °C [35]. It is a weak polyelectrolyte of pKa ca. 7.4, comprising of basic segments, that behave as proton acceptors in response to changes in the environmental pH values [36]. PDMAEMA’s cationic character along with its pH dependent hydration/dehydration characteristics, low cytotoxicity, high encapsulation capacity and low enough immunogenicity establish it as a promising vector for biomacromolecules delivery, such as nucleic acids and proteins, aiming at gene and protein delivery applications [37].

Reversible addition fragmentation chain transfer polymerization (RAFT) exhibits notable advantages in the synthesis of intelligent (smart) polymers, and random and block copolymers [38]. Firstly, it allows the synthesis of well-defined polymers of predetermined molecular characteristics [2,38,39,40]. Molecular weight and composition can be manipulated by fitting the polymerization conditions. Incorporation of a variety of monomers, multiplicity of molecular architectures, mild and manageable polymerization conditions, absence of catalysts to be removed after the end of polymerization and the potential for conducting the polymerization process in aqueous media are only a few beneficial features that RAFT polymerization provides [38,41,42].

In this work, we report on the synthesis of poly(2-(dimethylamino) ethyl methacrylate)-b-poly((oligo ethylene glycol methyl ether methacrylate) (PDMAEMA-*b*-POEGMA) double hydrophilic diblock copolymers by utilizing sequential RAFT polymerization [43] and their subsequent partial hydrophobic chemical modification by quaternization reaction with iodohexane and iodododecane to produce novel P(DMAEMA-co-Q_6_DMAEMA)-*b*-POEGMA and P(DMAEMA-co-Q_12_DMAEMA)-*b*-POEGMA random diblock copolymers, respectively (Q_6_ and Q_12_ prefixes present the number of carbon atoms that the attached alkyl chain on the PDMAEMA groups bears). The result of the partial chemical modifications is the construction of novel diblock copolymers where the first block contains hydrophilic and hydrophobic monomeric units that are distributed in a random fashion. The amine groups of the PDMAEMA block were partially modified with iodohexane or iodododecane in a predetermined percentage, in order to attach positive charges in some amine side groups [44,45,46] for subsequent bioapplications, and at the same time introduce long alkyl chains that will amplify the hydrophobic character of the polymeric system [44]. The DMAEMA monomer was chosen bearing in mind its responsiveness to pH and temperature changes, as well as its potential application to nanomedical fields. The OEGMA monomer was included due to ameliorated solubility and colloidal stability that oligo ethylene glycol side chains introduce, as well as the biocompatibility and low-cytotoxicity it exhibits [47]. The quaternization of the tertiary amine groups of PDMAEMA, using long alkyl halides as the quaternization factors to fabricate permanent cationic units along the polymer chain, has been investigated in a limited number of cases [44,48,49], while quaternization of PDMAEMA using methyl iodide (CH_3_I) is more intensively studied [50,51,52,53]. Cationic polymers are important in a wide range of applications such as antifouling, and drug, gene and protein delivery [54]. In particular, Κoufakis et al. reported on the synthesis of cationic polymeric brushes which display bactericidal character against both Gram-positive and Gram-negative bacteria strains when alkyl chains of less than six carbons atoms are grafted [49]. The PDMAEMA block was chosen not to be completely quaternized in order to maintain some pH and temperature responsiveness, while the incorporation of long alkyl chains will allegedly influence the response of all obtained modified random diblock copolymers to both stimuli [44,55]. Therefore, the self-assembly behavior of the partially quaternized random diblock copolymers was investigated upon heating and at varying pH of their aqueous solutions. The ameliorated hydrophobic character of the obtained partially quaternized random diblock copolymers established by the attachment of the long alkyl chains, establishes them also as potential nanocarriers for hydrophobic drugs. Thereupon the interactions of the quaternized random diblock copolymers with indomethacin and the resulting drug loaded nanoparticles were investigated with light scattering techniques (DLS, SLS, ELS) and infrared spectroscopy (ATR-FTIR). Indomethacin is a hydrophobic, anti-inflammatory drug commonly prescribed to diminish physical symptoms such as fever, ache, stiffness, and swelling from inflammation [56]. The pharmaceutical substance is expected to be encapsulated into the internal hydrophobic domains of the diblock nanostructures. The loading efficiency was determined by utilizing UV-Visible spectroscopy (UV-Vis), while release studies were conducted by applying ultrasound, demonstrating the on demand release of the drug.

## 2. Materials and Methods

### 2.1. Materials

Monomers 2-(dimethylamino)ethyl methacrylate (DMAEMA, 98%) and (oligo ethylene glycol)methacrylate (OEGMA) (average M_n_ = 475 g/mol, 9 ethylene oxide units) were purchased from Sigma Aldrich, Athens, Greece. Monomers were purified by passing through a column packed with inhibitor removers before polymerization. 2,2′-Azobis(isobutyronitrile) (AIBN) was purified by recrystallization from methanol. 4-Cyano-4-[(dodecylsulfanylthiocarbonyl)sulfanyl]pentanoic acid (CPD) as the CTA, 1-iodohexane (C_6_H_13_I. ≥98%), 1-iodododecane (C_12_H_25_I, 98%) 1,4-dioxane (≥99.8% pure), which was dried over molecular sieves before use, tetrahydrofuran (THF, ≥99.9% pure), and n-hexane (≥97%) were also purchased from Sigma Aldrich, Athens, Greece. Indomethacin (IND) was obtained from Fluka, Athens, Greece and used as received. Experimental details on the diblock copolymer synthesis are provided in the Supplementary Materials section.

### 2.2. Self-Assembly of the Partially Chemically Modified P(DMAEMA-co-Q_6_DMAEMA)-b-POEGMA and P(DMAEMA-co-Q_12_DMAEMA)-b-POEGMA Random Diblock Copolymers

The synthetic process of the PDMAEMA-*b*-POEGMA diblock copolymers and the partial quaternization of the PDMAEMA block in order to produce P(DMAEMA-co-Q_6/12_DMAEMA)-*b*-POEGMA random diblock copolymers is described in detail in the Supplementary Materials. Τwo protocols were followed in order to prepare stock solutions of the partially hydrophobically modified random diblock copolymers. The first one involves the direct dissolution of dry solid copolymers in distilled water. The second one concerns the initial dissolution of the dry solids in THF, the addition of the mixture in distilled water and then the evaporation of the organic solvent. Specifically, the first protocol includes the addition of the appropriate volume of distilled water of pH 7, in a specific quantity of copolymer’s dry solid. The resulted mixture remained for two hours at room temperature, then was heated up to 60 °C for three hours under stirring and then heating continued overnight up to 60 °C in an oven. Afterwards, the solution was left overnight at room temperature and studied the following day in order to be in thermodynamic equilibrium. By implementing this protocol, samples of concentration 5 × 10^−3^ g/mL (10 mL of water of pH 7 were placed in a vial containing 50 mg of dry solid) and 1 × 10^−3^ g/mL were prepared. The second protocol involves the dissolution of 10 mg of dry copolymer in 2 mL of THF and subsequently the injection of the resulted solution in distilled water of pH 7, under vigorous stirring. After two minutes of stirring, the latter mixture was heated until THF was evaporated and the final aqueous solution of 1 × 10^−3^ g/mL concentration was obtained. The final solution was kept at room temperature overnight and studied the following day. The pH studies were held by carrying out the same preparation process, regardless of the protocol that was followed in order to prepare the aqueous solutions. The variable pH protocol that was followed is described in the following. The next day a certain volume of each aqueous stock solution was taken in order to regulate the pH of the resulting solutions. 10 μL of 0.1 M HCl was added in order to tune the pH of the aqueous solution of c = 1 × 10^−3^ g/mL at pH 3, while 50 μL of 0.1 M HCl was added in order to tune the pH of the aqueous solution of c = 5 × 10^−3^ g/mL at pH 3. 50 μL of 0.1 M NaOH to tune the solution of c = 1 × 10^−3^ g/mL at pH 10 and 50 μL of 0.1 M NaOH to tune the solution of c = 5 × 10^−3^ g/mL at pH 10. The final solutions where the pH was regulated at 3 and 10, respectively, were left for 2 h before conducting the measurements, in order to achieve equilibrium. Light scattering techniques (dynamic light scattering/DLS, static light scattering/SLS and electrophoretic light scattering/ELS) were used to study the properties of all aqueous copolymer solutions. The aqueous solutions of the more hydrophobic derivatives of the PDMAEMA_42_-*b*-POEGMA_12_ precursor diblock were prepared only by using the organic solvent protocol (see Supplementary Materials).

### 2.3. Interaction Studies of P(DMAEMA-co-Q_6_DMAEMA)-b-POEGMA and P(DMAEMA-co-Q_12_DMAEMA)-b-POEGMA Random Diblock Copolymers with Indomethacin (IND)

#### 2.3.1. Indomethacin (IND) Loading Studies

Mixed nanoaggregates of the random diblock copolymers with indomethacin were prepared by using the following process. At first IND solutions and Q_6_, Q_12_ modified diblocks of quaternization degree 50% in THF (5 × 10^−3^ g/mL) were prepared. The IND concentration in THF was calculated according to the targeted encapsulated drug amount. The intended IND encapsulation degrees were 10 and 20% based on the total mass of the diblock utilized in each time. Subsequently, the two solutions were mixed in appropriate ratios, to vary the IND/copolymer mass ratio and the mixtures were injected fast in distilled water of pH 7 that was vigorously stirred. The latter mixture was heated until THF was evaporated and mixed IND/P(DMAEMA-co-Q_6/12_DMAEMA)-*b*-POEGMA solutions of final concentration of 1 × 10^−3^ g/mL were obtained. The mixed solutions were investigated by light scattering techniques. The IND loading efficiency was determined with the assistance of a calibration curve of IND in THF by a Perkin-Elmer, Lambda 19 spectrophotometer, USA at λ_max_ = 318 nm. The interactions between IND and partially quaternized samples were studied via ATR-FTIR spectroscopy (Bruker Optik, Ettlingen, Germany).

#### 2.3.2. IND Release Studies

The polymeric aggregates with the encapsulated IND were placed in dialysis bags of 3.5 kDa MWDO. Specifically, 10 mL of solution were added in the dialysis bag which was placed in 100 mL of water for injection and then put in a SOLTEC, SONICA 3300ETH-S3 ultrasonic bath. Amounts of the external aqueous solution were taken in defined time intervals and each time the aqueous solution was restored to its initial volume, so that the reservoir conditions remain constant. The IND percentage that was released at different time intervals, until 4 h, was assessed with the aid of a calibration curve of IND in THF by a Perkin-Elmer, Lambda 19 spectrophotometer, at λ_max_ = 318 nm. Initial studies conducted without the use of sonication bath indicated that IND could not be released from the aggregates in a period of 12 h.

### 2.4. Characterization Methods

A Waters size exclusion chromatography (SEC) instrument from Waters Technologies Corporation, Caguas, Puerto Rico, was utilized for the determination of the molecular weights and the molecular weight distributions of the synthesized PDMAEMA homopolymers and PDMAEMA-*b*-POEGMA precursor diblock copolymers. It was equipped with a Waters 1515 isocratic pump, a set of three μ-Styragel mixed bed columns (pore diameter between 10^2^ and 10^6^ Å) and a Waters 2414 refractive index (equilibrated at 40 °C). Breeze software was used for data acquisition and analysis. THF (containing 5% *v*/*v* triethylamine) comprised the mobile phase, at a flow rate of 1.0 mL/min, at 30 °C. Polystyrene standards of average molecular weights between 1200 and 152,000 g/mol and narrow molecular weight distributions were utilized for the calibration of the SEC set-up. In order to determine the molecular weights and the molecular weight distributions, the polymers were dissolved in THF at a concentration of 1 × 10^−3^ g/mL.

^1^H-NMR experiments were conducted on a Varian 300 (600MHz) spectrometer using Vjnmr software obtained from Scientific instruments, Palo Alto, California and tetramethylsilane (TMS) used as the internal standard in D_2_O. The chemical shifts are reported in the following.

A BI-DNDC Differential Refractometer from Brookhaven instruments Holtsville, New York, NY, United States was utilized for the determination of the dn/dc of the P(DMAEMA-co-Q_6_DMAEMA)-*b*-POEGMA and P(DMAEMA-co-Q_6_DMAEMA)-*b*-POEGMA random diblock copolymers, in solutions. The instrument calibration was conducted by measuring aqueous solutions of KCl at specific concentrations.

Dynamic light scattering (DLS) experiments were conducted on an ALV/CGS−3 compact goniometer system (ALV GmbH), composed of an ALV 5000/EPP multi-τ digital correlator with 288 channels and an ALV/SE-5003 light scattering electronics unit for stepper motor drive and limit switch control. A JDS Uniphase 22 mW He-Ne laser (λ = 632.8 nm) was utilized as the light source. All obtained by ALV GmbH, Langen (Hessen), Germany. Autocorrelation functions were analyzed by the cumulants method [57] and the CONTIN algorithm [58]. Before measurements, dust particles were removed from the solutions by filtration through 0.45 mm hydrophilic PVDF filters. The samples were put into 1 cm width quartz cells and measurements were carried out within the angular range of 30–150°. A Polyscience model 9102 bath, purchased from Polyscience, Illinois, USA, equipped with a circulator was used in order to regulate temperature inside the measuring cell. Measurements were conducted in the temperature range of 25 °C to 60 °C, in 5 °C steps. Static light scattering (SLS) experiments were conducted on the same instrument at angles 30–150° and from 25 °C to 55 °C temperature range. Toluene was used as the calibration standard. The R_g_/R_h0_ ratios were calculated by implementing the Guinier method to light scattering data.

Electrophoretic light scattering (ELS) studies were carried out on a ZetaSizer Nano series Nano ZS (Malvern Instruments Ltd., Malvern, United Kingdom), composed of a He-Ne laser at a wavelength of 633 nm and a fixed backscattering angle of 173°. The Henry approximation of the Smoluchowski equation [59] was used to analyze the obtained data, after equilibration of the polymer solutions at 25 °C and 50 °C. The recorded zeta-potential values are averages of 100 scans.

Optical absorption spectra of the P(DMAEMA-co-Q_6_DMAEMA)-*b*-POEGMA/IND and P(DMAEMA-co-Q_12_DMAEMA)-*b*-POEGMA/IND mixed solutions, IND solutions in THF and aqueous solutions of the released IND, were recorded on a Perkin-Elmer, Lambda 19 UV-Vis-NIR spectrophotometer, obtained from PerkinElmer Inc., USA. The measurements were carried out in the range of 200–450 nm.

ATR-FTIR measurements were conducted at room temperature in the spectral range 5000 to 500 cm^−1^ on a Bruker Equinox 55 Fourier transform instrument (Bruker Optik, Ettlingen, Germany), utilizing a single bounce attenuated total reflectance (ATR) diamond accessory (Dura-Samp1IR II by SensIR Technologies, Chapel Hill, North California). Background spectra were obtained by recording the clean and dry ATR diamond crystal surface against air and then subtracted from the sample spectrum. PDMAEMA_56_-*b*-POEGMA_86_ and P(DMAEMA_45_-b-Q_6_DMAEMA_11_)-*b*-POEGMA_86_ diblock copolymers were measured at their solid state, after the purification process was completed, in order to confirm the modified chemical structure of the diblock copolymer precursor was accomplished. In order to detect any interactions between the random diblock copolymers with IND, the aqueous solutions of the random diblock copolymers and the mixed nanoassemblies with IND were used. In these cases, water was evaporated by utilizing nitrogen so that the water absorption peaks would not intervene with the peaks that would show the interactions between IND and the random diblock copolymers. Afterwards, solid IND was measured in order to assign the presence of the new peaks at the ATR spectra of the mixed nanossemblies to the characteristic functional groups of IND. Additionally, 64 interferograms were collected for every single spectrum with a resolution of 4 cm^−1^ to attain the better signal/noise ratio.

## 3. Results and Discussion

### 3.1. Synthesis of P(DMAEMA-co-Q_6/12_DMAEMA)-b-POEGMA Chemically Modified Random Diblock Copolymers

**Scheme 1:**
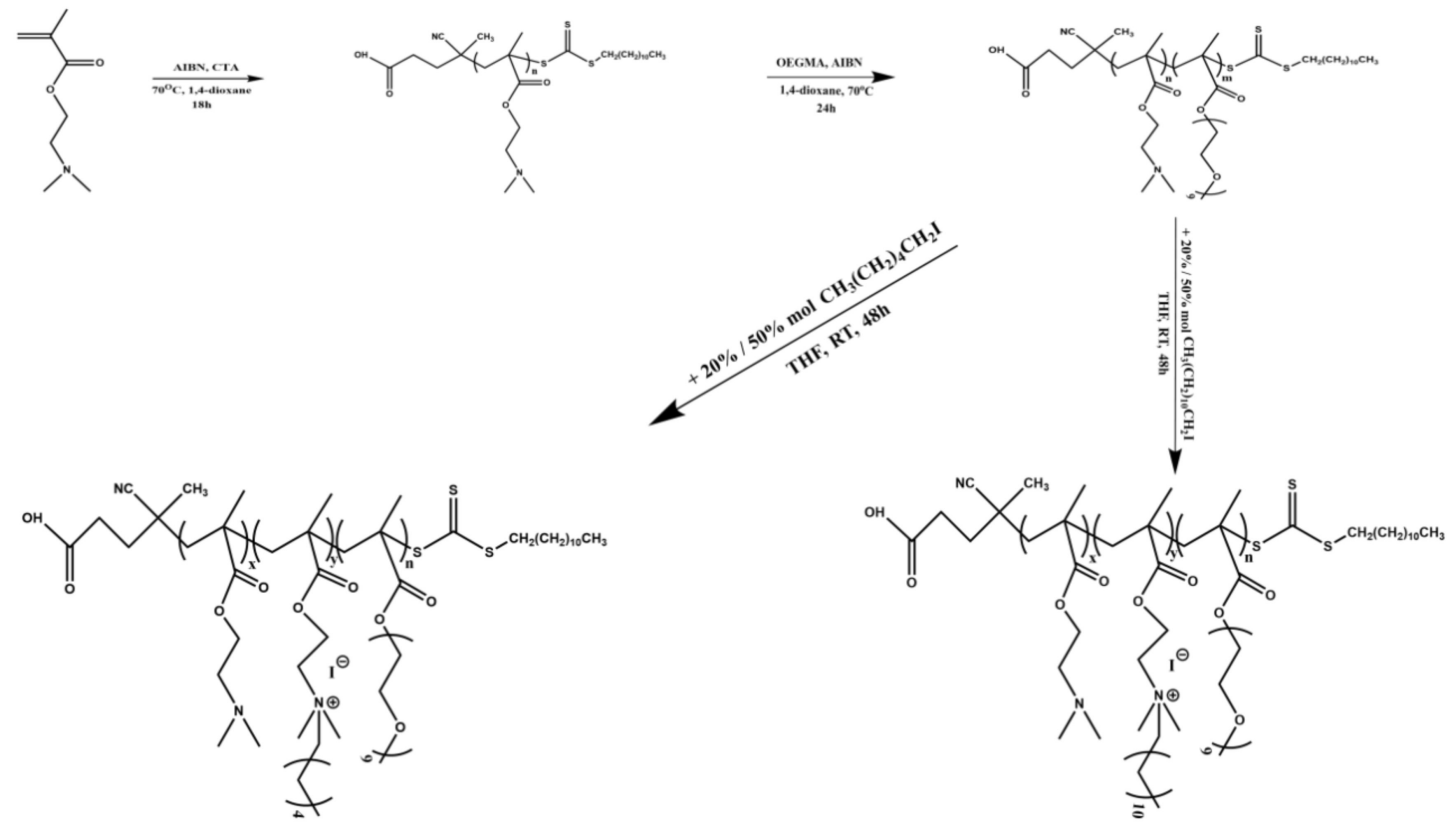
Synthesis route to partially chemically modified P(DMAEMA-co-Q_6_DMAEMA)-*b*-POEGMA and P(DMAEMA-co-Q_12_DMAEMA)-*b*-POEGMA random diblock copolymers, via quaternization reactions of the PDMAEMA-*b*-POEGMA precursors, that were synthesized by RAFT, using iodohexane and iodododecane in different molar ratios.

**Figure 1:**
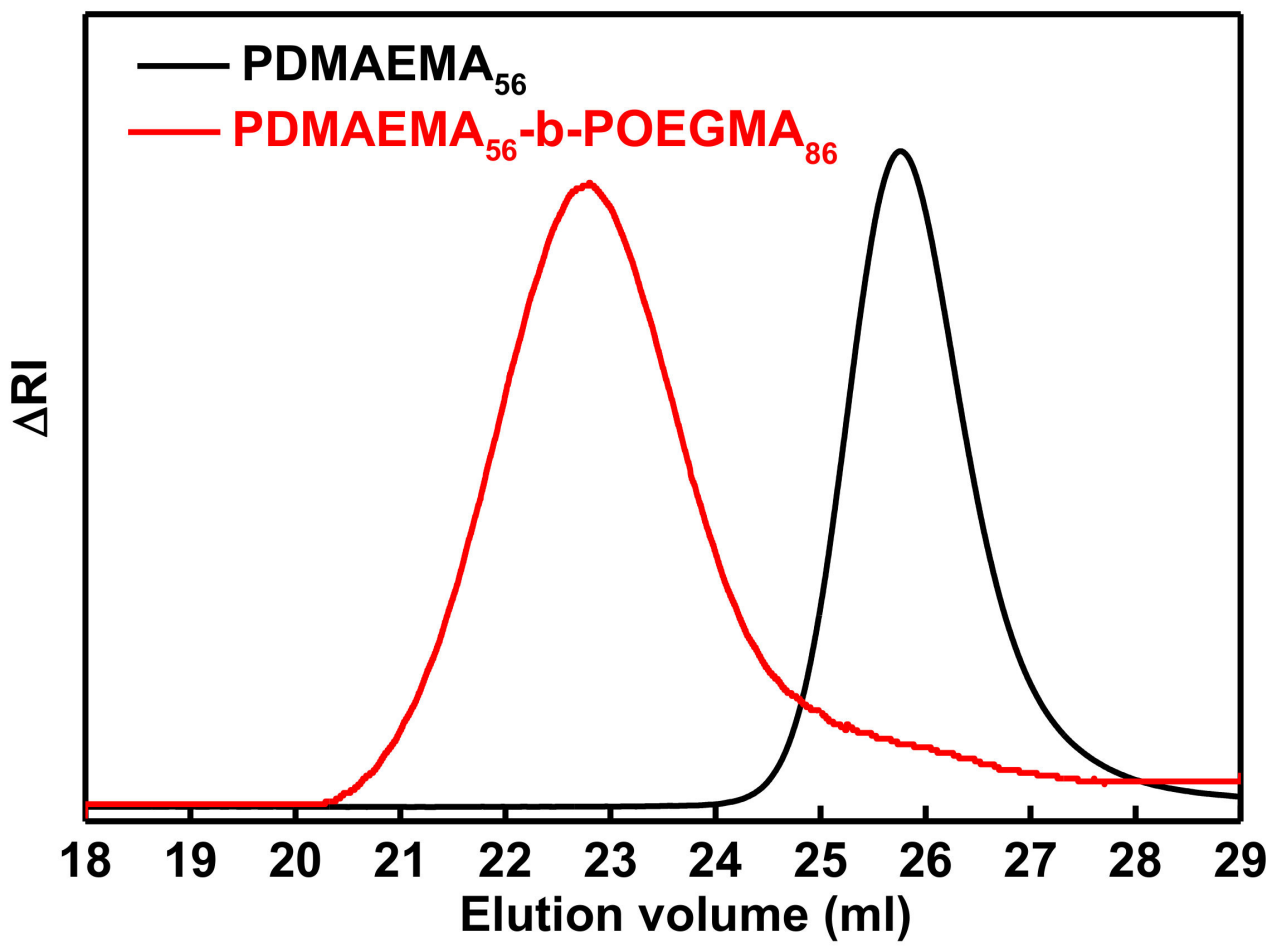
SEC chromatograms of PDMAEMA_56_ (first block) and PDMAEMA_56_-*b*-POEGMA_86_ (final diblock copolymer).

Double hydrophilic, pH and temperature responsive PDMAEMA_56_-*b*-POEGMA_86_ and PDMAEMA_42_-*b*-POEGMA_12_ were successfully synthesized by sequential RAFT polymerization. The combination of monomers towards synthesis of block copolymers via RAFT has been reported before [43]. PDMAEMA_56_ and PDMAEMA_42_ homopolymers were utilized as the macro-CTAs for the synthesis of PDMAEMA_56_-*b*-POEGMA_86_ and PDMAEMA_42_-*b*-POEGMA_12_ diblock copolymers respectively. The synthetic process is depicted in Scheme 1. CPD was chosen as the CTA based on its compatibility with these type of monomers provided by studies performed at our lab [52]. SEC measurements were made after each polymerization step to determine the successful polymerization of each block, the molecular weights and molecular weight distributions of PDMAEMA homopolymers and their resulting diblock copolymers. As it can be observed in Figure 1, where SEC chromatograms of the PDMAEMA_56_ and the resulted PDMAEMA_56_-*b*-POEGMA_86_ are depicted, the molecular weight is increased after the incorporation of the POEGMA block, which is confirmed by the shift of the molecular weight distribution to lower elution volumes. Overall, SEC chromatograms (Figure 1) revealed the efficiency of the synthetic procedure followed and resulted in the formation of rather well-defined PDMAEMA_56_-*b*-POEGMA_86_. The molecular weight distribution is adequately narrow and relatively symmetrical, even though a small tail exists indicating the almost complete re-initiation and consumption of the OEGMA units for producing the POEGMA block. Similar conclusions are extracted from the SEC chromatograms of PDMAEMA_42_-*b*-POEGMA_12_ diblock copolymer. Molecular weights and polydispersity indexes of all synthesized polymers are presented in Table 1. The molecular weight distributions is in accordance with the principles of RAFT polymerization. 

The molecular characteristics of both PDMAEMA-*b*-POEGMA diblock copolymers are presented in Table 1. ^1^H-NMR comparative spectra of the PDMAEMA_42_ and PDMAEMA_42_-*b*-POEGMA_12_ can be found in SI (Figure S2).

Post-polymerization partial quaternization of the DMAEMA segments of the PDMAEMA-*b*-POEGMA precursors was achieved towards production of novel chemically modified P(DMAEMA-co-Q_6_DMAEMA)-*b*-POEGMA and P(DMAEMA-co-Q_12_DMAEMA)-*b*-POEGMA random diblock copolymers by using two alkyl halides of different alkyl chain length. By implementing partial quaternization of DMAEMA segments, only part of the tertiary amine groups of DMAEMA are converted into their quaternary analogues. Consequently, the synthesis of novel amphiphilic random diblock copolymers was accomplished by the method described above. PDMAEMA a weak cationic polyelectrolyte that after quaternization process takes place it transforms into a partially strong cationic polyelectrolyte [44]. This conversion is of great importance as a number of cationic charges, defined by the quaternization degree, are permanently present onto the amine groups and are randomly distributed along the modified block. Moreover, the presence of cationic groups allows the utilization of the present copolymers in gene delivery applications and increases the antifouling ability of the copolymers [49]. By modifying only part of the total amount of amino groups, responsiveness of the diblocks to pH and temperature variations is expected to be altered but not to be completely absent [46,60]. The addition of long alkyl chains of different lengths is another interesting characteristic that is worth investigating as they impart amphiphilic character to the random diblock and as a result, they affect the self-assembly properties [44]. In addition, the presence of long hydrophobic groups should affect the pH and temperature-induced behavior of the formed nanoassemblies. It should be noted that the existence of POEGMA block would assist in surpassing solubility obstacles and provide colloidal stability in the aqueous solutions of the partially quaternized diblock copolymers, as well as increased biocompatibility. The effect of the POEGMA composition on the self-organization behavior of the nanostructures is investigated below by light scattering techniques. PDMAEMA-*b*-POEGMA precursors were modified at 20 and 50% molar ratio using 1-iodohexane 7(C_6_H_13_I) and 1-iodododecane (C_12_H_25_I) (C_6_H_12_I moles / PDMAEMA moles = 0.2 and 0.5, C_12_H_25_I moles / PDMAEMA moles = 0.2 and 0.5). Specifically, by partial quaternization of the PDMAEMA_56_-*b*-POEGMA_86_ precursor four different chemically modified diblock copolymers were obtained, two with alkyl hydrophobic chains of six carbons atoms and two with alkyl hydrophobic chains of twelve carbon atoms. The two copolymers containing alkyl hydrophobic chains of the same carbon atoms differ in the number of the alkyl chains attached to the amine groups of the DMAEMA residues, i.e., in the degree of quaternization. The same scheme was followed for the partial quaternization of PDMAEMA_42_-*b*-POEGMA_12_ precursor diblock. In summary, eight novel partially hydrophobically modified diblock copolymers were obtained, that differ in their molecular characteristics. 

**Figure 2:**
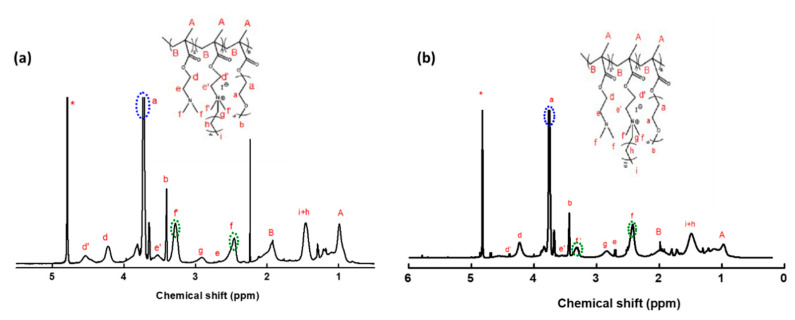
^1^H-NMR spectra for (**a**) P(DMAEMA-co-Q_6_DMAEMA)-*b*-POEGMA and (**b**) P(DMAEMA-co-Q_12_DMAEMA)-*b*-POEGMA in D_2_O.

Figure 2a displays the ^1^H-NMR spectrum for the P(DMAEMA-co-Q_6_DMAEMA)-*b*-POEGMA random diblock copolymer that derived from the partial quaternization of PDMAEMA_42_-*b*-POEGMA_12_, at nominal degree of quaternization 50% using 1-iodohexane. The characteristic spectral peaks of both the DMAEMA and Q_6_DMAEMA segments are observed at 2.42 ppm (-NH(CH_3_)_2_-) and 3.35 ppm (-N^+^((CH_3_)_2_(CH_2_))-) respectively. The degree of quaternization of the diblock copolymer was estimated by determining the integrals of the peaks at 2.42 ppm and at 3.35 ppm, which correspond to the –CH_3_ protons of the tertiary amines of the DMAEMA residues and the -CH_3_ protons attached to the quaternary amino groups of the Q_6_DMAEMA units, respectively. The degree of quaternization was determined to be 48%. The same methodology was followed in order to determine the quaternization degree of the P(DMAEMA-co-Q_12_DMAEMA)-*b*-POEGMA random diblock copolymer (quaternization agent: 1-iodododecane), derived from the partial quaternization of PDMAEMA_42_-*b*-POEGMA_12_ that is depicted in Figure 2b. The degree of polymerization was determined to 20%, which completely agrees with the theoretical one using 1-iodohexane. Generally, the degrees of quaternization found by NMR were in almost complete accordance with the theoretical values calculated by using the molar ratio of the alkyl halide to the DMAEMA monomeric units [44]. Moreover, in each case of the P(DMAEMA-co-Q_6_DMAEMA)-*b*-POEGMA (Figure 2a) the weight composition of the POEGMA block was determined by integrating the peak of the methylene proton groups at 3.74 ppm. The same methodology was followed for each one of the remaining modified random diblock copolymers. The different quaternization degrees when using the same halide, along with the weight composition of PDMAEMA homopolymers, PDMAEMA-*b*-POEGMA precursor diblocks and the resulted eight partially modified random copolymers are presented in Table 1. Further confirmation of the modified chemical structure of the diblock copolymer precursor was accomplished by utilizing ATR-FTIR measurements. A representative example of comparative spectra of diblock precursor and its analogue can be found in SI (Figure S3).

### 3.2. Self-Assembly of the P(DMAEMA-co-Q_6/12_ DMAEMA)-b-POEGMA Random Diblock Copolymers in Aqueous Media

Studies on the ability of the resulted eight novel P(DMAEMA-co-Q_6/12_ DMAEMA)-*b*-POEGMA diblocks to self-assemble in aqueous media were conducted. In contrast to the double hydrophilic PDMAEMA-*b*-POEGMA precursor diblocks, their partially quaternized analogues are expected to exhibit amphiphilic character due to the incorporation of long hydrophobic chains. Partial quaternization reaction results in cationic units and the formation of the P(DMAEMA-co-QDMAEMA) random block. Subsequently, the amphiphilic character of the random diblock copolymers should define the formation of nanoaggregates. Based on the nature and sequence of the blocks, the P(DMAEMA-co-QDMAEMA) random block should occupy the core-like region and the POEGMA block the corona of the aggregates. When inserted in aqueous media, it is expected that the presence of the hydrophobic groups would result in intramolecular interactions of the pendant groups leading to hydrophobic nanodomains [46] and thus the formation of nanoaggregates, whose morphological features will depend on the hydrophobic chain length and the quaternization degree. Due to close spatial vicinity of hydrophilic and hydrophobic groups the inner regions of the aggregates are expected to include hydrophobic microdomains but also water due to both the hydrophilic character of the DMAEMA units at pH = 7 and T = 25 °C and that of the cations attached to the quaternary amines of the modified QDMAEMA residues. Subsequently, the interface between corona and core may not be so well-defined as in normal core-shell micelles formed by traditional diblock copolymers. The morphology, mass and size of the nanoassemblies is expected to be affected by the length of the alkyl chain and the quaternization degree. The nanostructures formed from the self-assembly of the random diblock copolymers that contain alkyl chains of twelve carbon atoms are anticipitated to present more hydrophobic character. This hypothesis is based on the existence of longer hydrophobic chains and thus amplification of hydrophobic interactions that will result to lower interaction with water molecules [61]. Furthermore, the coexistence of DMAEMA units along with QDMAEMA residues will affect the response of DMAEMA units to pH and temperature changes. As a result, further aggregation or disaggregation may occur due to protonation or deprotonation of the amine groups of the DMAEMA segments, induced by altering the solution pH. In case of temperature increase, the phase transition from a hydrated/hydrophilic state to dehydrated/more hydrophobic state is expected to occur at lower temperature values than the reported LCST of the PDMAEMA homopolymer. This particular behavior is assigned to the presence of entirely hydrophobic molecules that will assist to the dehydration of the polymeric system due to amplification of the hydrophobic interactions [61]. Light scattering techniques were utilized to examine the self-assembly of the modified diblock copolymers in aqueous solutions as a response to pH and temperature changes. DLS was utilized to acquire the hydrodynamic radius (R_h_, size indicator) and the size polydispersity index (PDI) of the formed nanoassemblies. PDI is extracted by using the cumulant method, while R_h_ is obtained by the CONTIN algorithm. SLS was applied to obtain the average scattering intensity values (I) (a parameter that primarily indicates alterations in mass of the aggregates) and the R_g_/R_h0_ ratios (in combination with DLS) and for detecting the pH response of the polymer chains. ELS was also conducted in order to define the ζ-potential values and overall surface charge of the nanoassemblies at varying pHs, i.e., at neutral, acidic and basic pH and at two temperatures i.e., 25 °C and 45 °C.

#### 3.2.1. pH Effects on the Self-Assembly Behavior of the P(DMAEMA-co-Q_6_/_12_DMAEMA)-*b*-POEGMA Random Diblock Copolymers

Taking into account, the sequence of the diblock copolymers, the nature of the monomers and their functionality, the distribution of distinct monomeric units in a random fashion within one block, the study of pH and temperature effects on the aqueous solutions of the random diblock copolymers seemed mandatory. In order to examine, the pH-response of the aqueous solutions, the investigations were held at three different pH values, namely at pH = 7 (neutral), 3 (acidic) and 10 (basic), representative of the entire pH range. For the preparation of the aqueous solutions at pH = 7 and T = 25 °C, two protocols were used. The first one includes the addition of distilled water at a vial containing the dry solid and then heating the mixture to 60 °C, until dissolution occurs. The measurements were held at T = 25 °C, after equilibrium was achieved (see Section 2.4). The second one includes the dissolution of the dry solid at THF, its subsequent mixture with distilled water and afterwards the evaporation of the organic solvent. The solution was examined at T = 25 °C, after equilibrium was accomplished (see Section 2.2). The choice to include two preparation protocols was based on the possible effects of each protocol on the morphology of the nanoaggregates and their colloidal stability over time, since nanoparticles formed using organic solvents are smaller in size and more thermodynamically stable [12,62]. The self-assembly characteristics upon pH-variations were compared according to the preparation protocol, matching nominal quaternination degree and nature of alkyl chains for the derivatives resulted from the partial quaternization of the PDMAEMA block of the same precursor block copolymer. The concentrations of the aqueous solutions of the analogues derived from the partial quaternization of the PDMAEMA_56_-*b*-POEGMA_86_, i, prepared by the direct dissolution in the aqueous medium were 1 × 10^−3^ g/mL and 5 × 10^−3^ g/mL. Below, discussion over the pH-response of the Q_6_ and Q_12_ derivatives of the first precursor of the same theoretical quaternization degree, when the preparation protocol of direct dissolution in aqueous media was used, is conducted. 

##### pH-Response of P(DMAEMA_45_-co-Q_6_DMAEMA_11_)-*b*-POEGMA_86_ and P(DMAEMA_44_-co-Q_12_DMAEMA_12_)-*b*-POEGMA_86_ for the Case of Direct Dissolution in the Aqueous Medium

**Figure 3:**
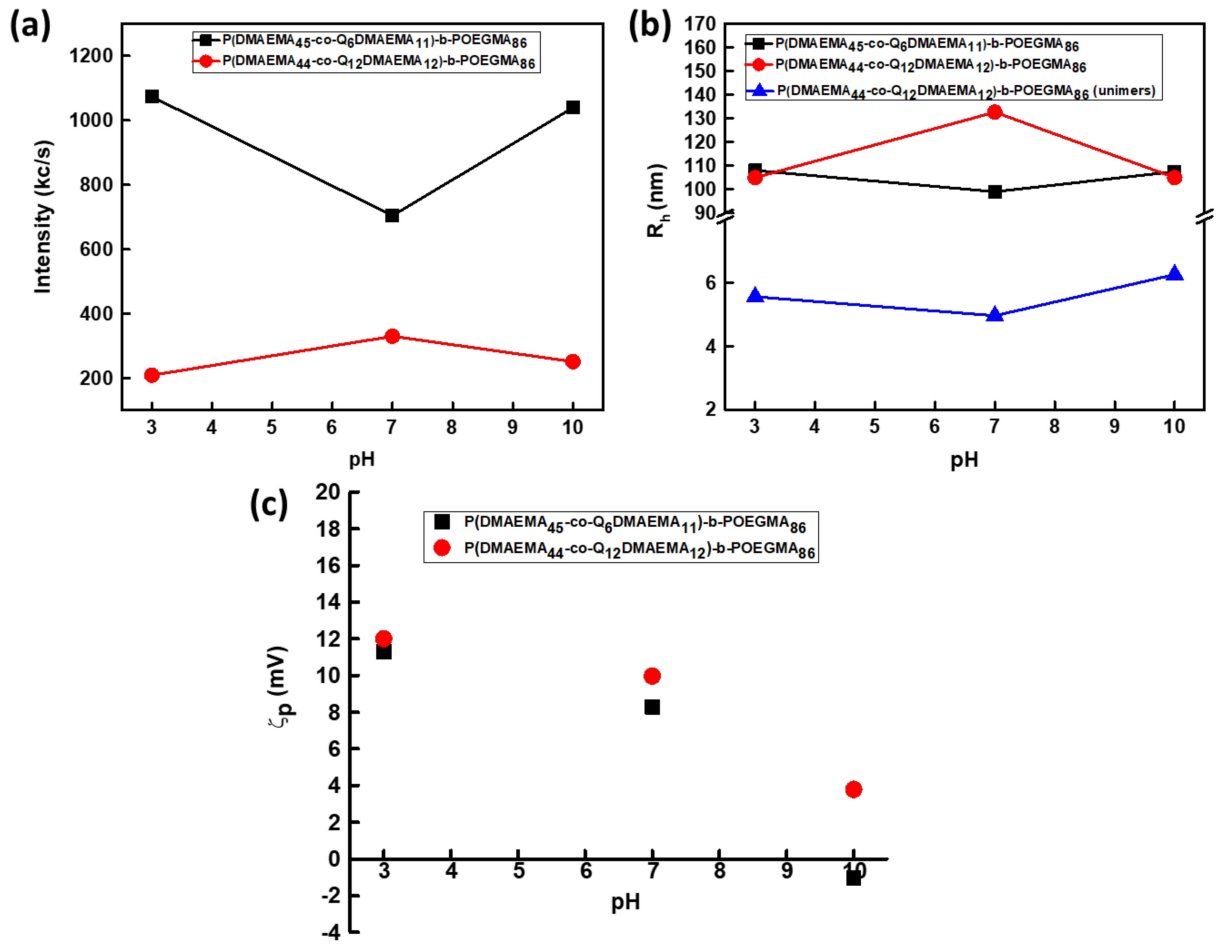
Dependence of (**a**) scattering intensity (I), (**b**) hydrodynamic radius (R_h_) and (**c**) ζ-potential (ζp) of the P(DMAEMA_45_-co-Q_6_DMAEMA_11_)-*b*-POEGMA_86_ and P(DMAEMA_44_-co-Q_12_DMAEMA_12_)-*b*-POEGMA_86_ aqueous solutions upon pH-variations, when the preparation protocol utilized was the direct dissolution in aqueous medium (c = 5 × 10^−3^ g/mL).

**Figure 4:**
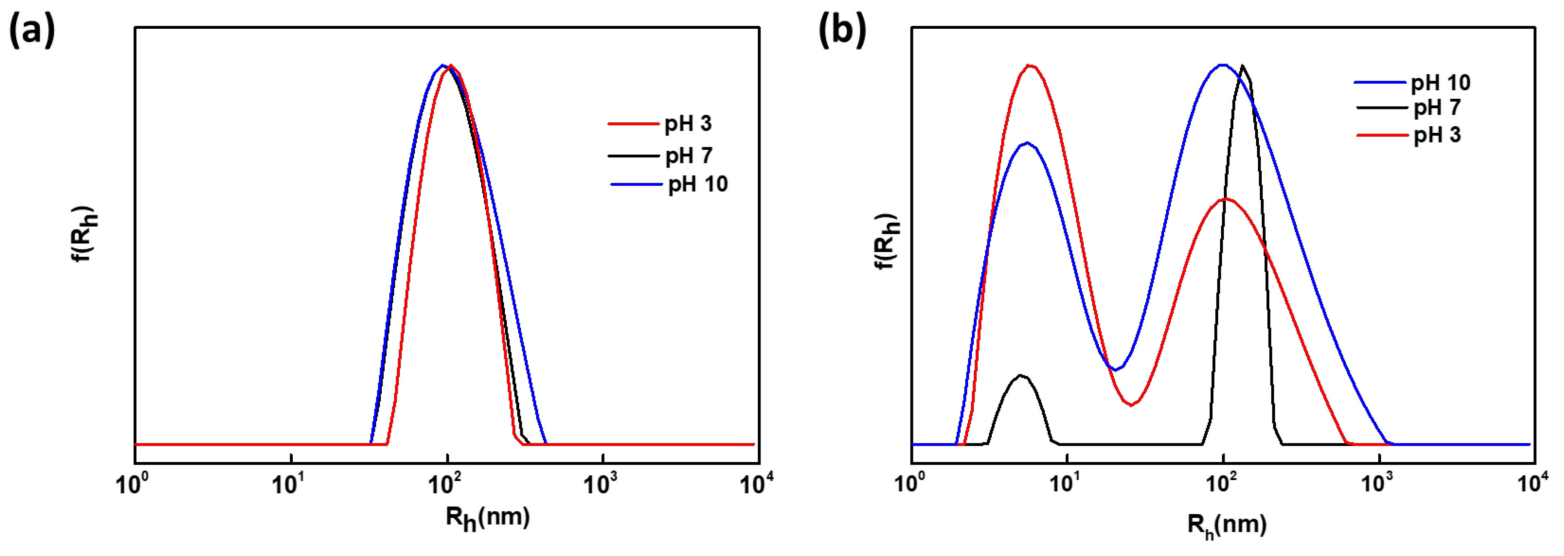
Size distributions from CONTIN analysis, at pH 7, 3 and 10, obtained by measurements held at 90°, for the aqueous solutions of: (**a**) P(DMAEMA_45_-co-Q_6_DMAEMA_11_)-*b*-POEGMA_86_ and (**b**) P(DMAEMA_44_-co-Q_12_DMAEMA_12_)-*b*-POEGMA_86_.

According to Figure 3a the scattering intensity of the Q_6_ diblock is considerably higher than the one of Q_12_ diblock at pH = 7, indicating the formation of nanoparticles of smaller mass when 1-iodododecane was used as the quaternization agent. This phenomenon is correlated to the existence of fewer but longer hydrophobic chains, resulting to initial aggregation due to a greater amplification of hydrophobic interactions and the formation of nanoaggregates of smaller mass. This behavior is also fully supported by the aggregation numbers (N_agg_) obtained for both samples by the SLS technique N_agg_ of the Q_6_ sample with quaternization degree of 19%, at pH 7 was determined to be 55 (±2.75) and 37 (±1.85) for the Q_12_ samples with degree of quaternization 20%). Moreover, based on the observation of the hydrodynamic radius versus pH variations, the nanoaggregates formed from the self-assembly of Q_12_ sample (R_h_ = 133 ± 6.65 nm), at pH = 7, are of higher dimensions than the ones of Q_6_ samples (R_h_ = 99 ± 4.95 nm). This may be also a result of better packing of the Q_6_ aggregates due to the shorter alkyl chains. Moreover, another population of smaller size is observed in the case of sample Q_12_, that is assigned to the presence of unimers (R_h_ = 5 ± 0.25 nm). Both observations are also supported by the lower scattering intensity values, the participation of fewer polymeric chains in the nanoassemblies formation and the smaller aggregation number. The coexistence of unimers and aggregates in the case of sample Q_12_ and the presence of only one population in the case of Q_6_ sample, at pH = 7, are shown in the size distribution from CONTIN analysis, displayed in Figure 4.

As far as, the pH response is concerned, both Q_6_ and Q_12_ diblocks are responding to variations of pH but quite differently. In the case of Q_6,_ at pH = 3, further aggregation occurs taking into consideration the increase in the scattering intensity compared with that at pH = 7, along with the increase of the hydrodynamic radius which at pH = 3 was determined at 108 ± 5.4 nm. This behavior is not the anticipated one, as shifting from neutral pH to acidic would provoke protonation of the amine groups of the DMAEMA units and subsequent disaggregation of the nanostructures. However, the complex morphology of the aggregates maybe accountable for the observed behavior. Increased aggregation may result by the presence of long alkyl chains which surround the amino groups, making them less accessible to hydrogen ions. Aggregation number also supports this conclusion (N_agg_ = 83 ± 4.2). When the pH turns 10 from 7, the scattering intensity and the hydrodynamic radius increase (to R_h_ = 104 ± 5.2 nm) due to further aggregation. The deprotonation of the amino groups at pH 10, provokes further aggregation phenomena. The latter are enhanced by the hydrophobic interactions from the presence of alkyl chains. The aggregation number also is increased at pH = 10. In the case of Q_12_ diblock, a pH-dependent behavior is observed. At pH = 3, based on the decrease in both scattering intensity and R_h_ values (R_h_= 150 ± 7.5 nm), the protonation of the amino group of the DMAEMA segments results in some splitting of the nanoaggregates. Aggregation number (N_agg_ = 23) is in accordance with the above analysis. Unimers (R_h_ = 5 ± 0.25 nm) also exist at pH = 3 and, based on the size distribution from CONTIN analysis (Figure 4b), the number of unimers is increased compared with that at pH = 3. At pH = 10, the scattering intensity is decreased from that at pH = 7 accompanied with a decrease in R_h_ value (R_h_ = 107 ± 7.5 nm), foreshadowing disaggregation phenomena. The aggregation number determined (N_agg_ = 30 ± 1.5) is in agreement with the above observation. From CONTIN analysis the existence of both unimers (R_h_ = 6 ± 0.3 nm) and aggregates is shown. The latter behavior was not expected, as deprotonation of the amino groups would generate further aggregation. Trying to explain this kind of response, one can reach to the conclusion that the nanoassemblies at pH 10 are not thermodynamically stable, taking also into consideration the high PDI value and the wide size distribution observed. The above hypothesis also applies to the nanoassemblies formed at pH = 3. The high PDI values for the Q_12_ sample, at all three pHs can be explained by the coexistence of two distinct populations (Figure 4b) and the random nature of the (PDMAEMA_44_-co-Q_12_DMAEMA_11_) block. The physicochemical characteristics of the diblocks at the aforementioned solution conditions are summarized in Table 2.

Combination of DLS and SLS techniques was used in order to determine the R_g_/R_h0_ ratios at all three pH values as this parameter provides more information about the morphology of the nanostructures [63,64]. As far as the 1Q_6_ diblock is concerned, the R_g_/R_h0_ values are in the range 0.65 to 0.80. According to existing literature R_g_/R_h0_ values in this range indicate the presence of nanostructures of spherical geometry with a relatively compact structure [63,64]. Τhe surface charge of both samples as a function of pH changes (Figure 3c), was monitored by ELS technique. At pH = 7 1Q_6_ diblock presents ζ-potential of +8.29 (±0.4) mV, while 1Q_12_ of +9.98 (±0.5) mV. Both values are in accordance with the permanent cationic charges induced by partial quaternization, combined with the nature of the random diblock copolymers that promotes the not well-defined interface between corona and inner hydrophobic region and the partial protonation of the DMAEMA segments. At pH = 3, the ζ-potential values are +11(±0.55) mV and +12 (±0.6) mV accordingly. At pH = 10 the ζ-potential of the 1Q_6_ diblock is −1 (±0.05) mV due to the deprotonation of the amino groups of the DMAEMA segments. However, the ζ-potential value of the 1Q_12_ is slightly positive (+3 ± 0.15 mV) at pH = 10 but within the anticipated range. 

##### pH-Response of P(DMAEMA_30_-co-Q_6_DMAEMA_26_)-*b*-POEGMA_86_ and P(DMAEMA_28_-co-Q_12_DMAEMA_28_)-*b*-POEGMA_86_ Solutions from the Direct Dissolution in the Aqueous Medium

**Figure 5:**
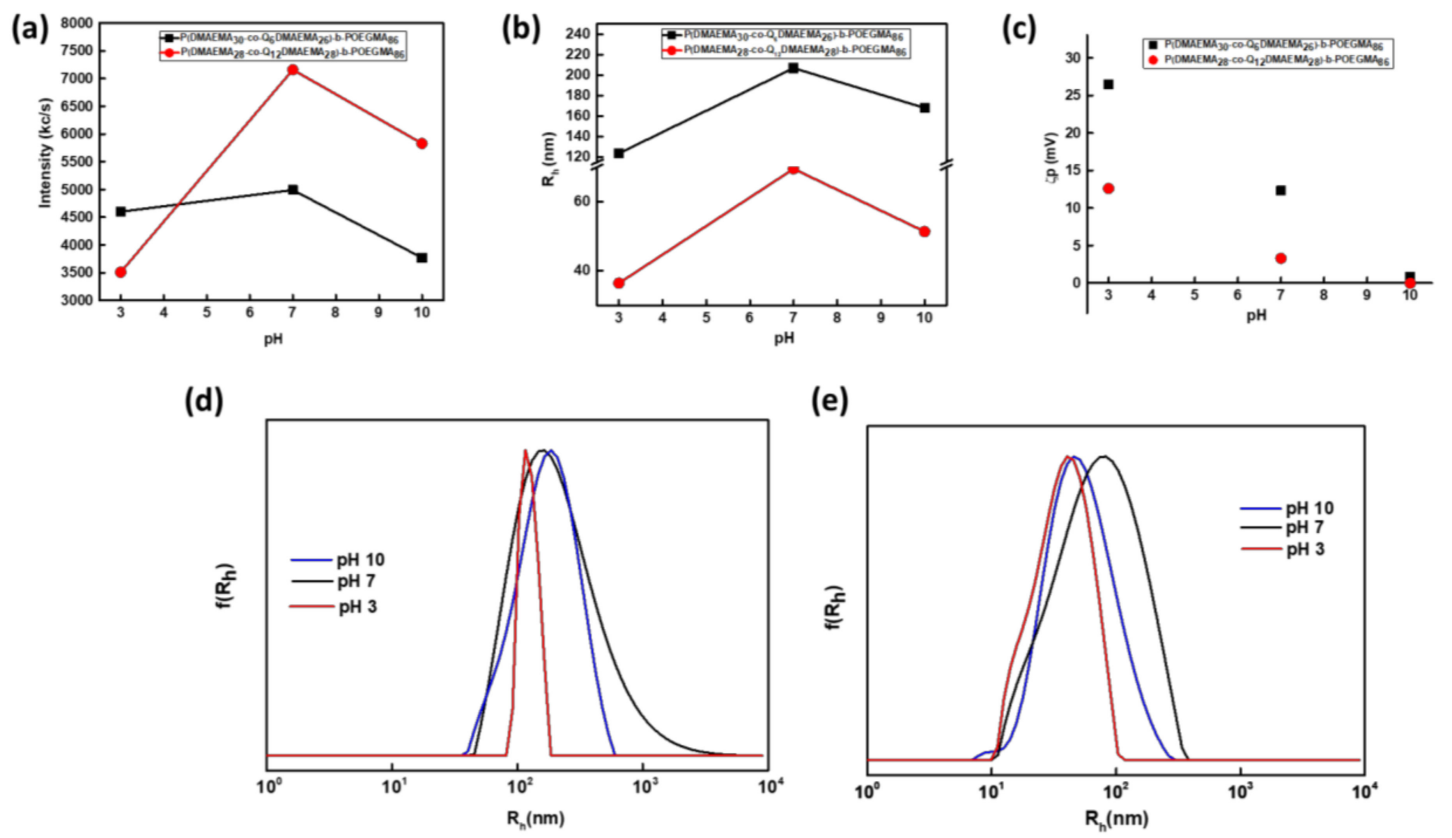
Dependence of (**a**) scattering intensity (Ι), (**b**) hydrodynamic radius (R_h_) and (**c**) surface charge (ζ_p_) for the P(DMAEMA_30_-co-Q_6_DMAEMA_26_)-*b*-POEGMA_86_ and P(DMAEMA_28_-co-Q_12_DMAEMA_28_)-*b*-POEGMA_86_ aqueous solutions on pH-variations and size distribution from CONTIN analysis obtained at 90°, at pH 7, 3 and 10 for (**d**) P(DMAEMA_30_-co-Q_6_DMAEMA_26_)-*b*-POEGMA_86_ and (**e**) P(DMAEMA_28_-co-Q_12_DMAEMA_28_)-*b*-POEGMA_86._ The preparation protocol was the direct dissolution in aqueous medium and the concentration of the solution 5 × 10^−3^ g/mL.

Accordingly, diblocks 2Q_6_ and 2Q_12_ were compared based on the response they present to pH variations. Based on the scattering intensity recorded as a function of pH (Figure 5), the self-assembly behavior at pH = 7 (before changing the solution pH), resulted in the formation of nanoaggregates of higher mass in the case of 2Q_12_ compared to the 2Q_6_ case. This latter behavior is assigned mostly in the increase in the quaternization degree of the diblocks and the formation of aggregates. This conclusion is fully supported by the recorded aggregation number (at pH 7 N_agg_ for 2Q_6_ is 65 (±3.3) and for 2Q_12_ is 110 (±5.5)). The reversed tendency is observed as far as the size of the nanoparticles is concerned. Specifically, the R_h_ value of 2Q_12_ at pH 7 is 69 nm (±3.5 nm) and for 2Q_6_ is 206 nm (±10 nm). Due to the higher hydrophobic character of 2Q_12_, more compact aggregates are formed. Moreover, for the 2Q_12_ diblock the absence of unimers is observed, in contrast to the 1Q_12_ diblock as the amplified hydrophobic character induces the participation of all polymeric chains in the formation of nanoaggregates. 

For the 2Q_12_ diblock when the solution pH changes from 7 to 3, the deprotonation of the amino groups of the DMAEMA residues results in disaggregation which is designated by the abrupt decrease in scattering intensity, size (R_h_ = 36 ± 1.8 nm) and aggregation number (N_agg_ = 45 ± 2.25). A non-expected behavior is observed when the solution pH changes to 10, where disaggregation occurs, even though further aggregation due to the deprotonation of the tertiary amines of DMAEMA was a more possible scenario. This observation is supported by the values of all the physicochemical parameters studied (at pH = 10, R_h_ = 51 ± 2.6 nm and N_agg_ = 75 ± 3.8). This behavior may be assigned to the peculiar morphology of the formed nanoaggregates or to the fact that they are not in thermodynamic equilibrium. For the 2Q_6_ diblock, a similar behavior, has been monitored but not of the same magnitude. These physicochemical quantities are displayed in Table 3. The similar behavior at pH 10 for both samples points to the conclusion that the increase of pH and thus amplification of the hydrophobic character of the chains induces the formation of thermodynamically unstable nanostructures. Size distributions from CONTIN analysis confirm the existence of one kind of population for both diblocks at all pHs. For 2Q_6_ diblock the size distribution peaks are rather broad and not so symmetrical, except for the pH = 3 case which is relatively narrow and symmetrical, with a lower PDI value (see Table 3). For the 2Q_12_ diblock the absence of unimers is confirmed. 

R_g_/R_h0_ values obtained after extrapolation at zero angle, at all cases, indicate the formation of nanoaggregates of spherical shape with rather compact inner structure. 2Q_6_ diblock exhibits higher surface charge than 2Q_12_. The latter fact may be attributed to the configuration of the polymeric chains that comprise the nano-structures. Apparently, the cationic components are located in the particle surface. The higher ζ_p_ values at pH 3 and the lower values at pH 10 (in comparison with the ones at pH 7) are expected to the protonation/deprotonation equilibria of DMAEMA segments, respectively.

It is worth mentioning that when the concentration of the aqueous solution was 1 × 10^−3^ g/mL for all four copolymers derived from the PDMAEMA_56_-*b*-POEGMA_86_ precursor, the scattering intensity was below 30 kc/s (±1.5) indicating that polymeric chains are actually molecularly dissolved in the aqueous medium. Therefore higher concentrations are eeded for the formation of aggregates in these polymeric systems. 

##### pH-Response of P(DMAEMA_30_-co-Q_6_DMAEMA_26_)-*b*-POEGMA_86_ and P(DMAEMA_28_-co-Q_12_DMAEMA_28_)-*b*-POEGMA_86_ Using the Organic Solvent Protocol

In this section, the results from the aqueous solutions of 2Q_6_ and 2Q_12_ samples as a function of pH changes, prepared by using the organic solvent (THF) solution preparation protocol, are discussed. The choice over studying the samples of about 50% quaternization degree was based on the fact that the increased hydrophobic character should induce the formation of well-defined nanoparticles with ameliorated features and enhanced pH-response. The use of this protocol offers the prospect of formation of nanoparticles closer to equilibrium [12]. The concentration of the investigated aqueous solutions was 1 × 10^−3^ g/mL.

**Figure 6:**
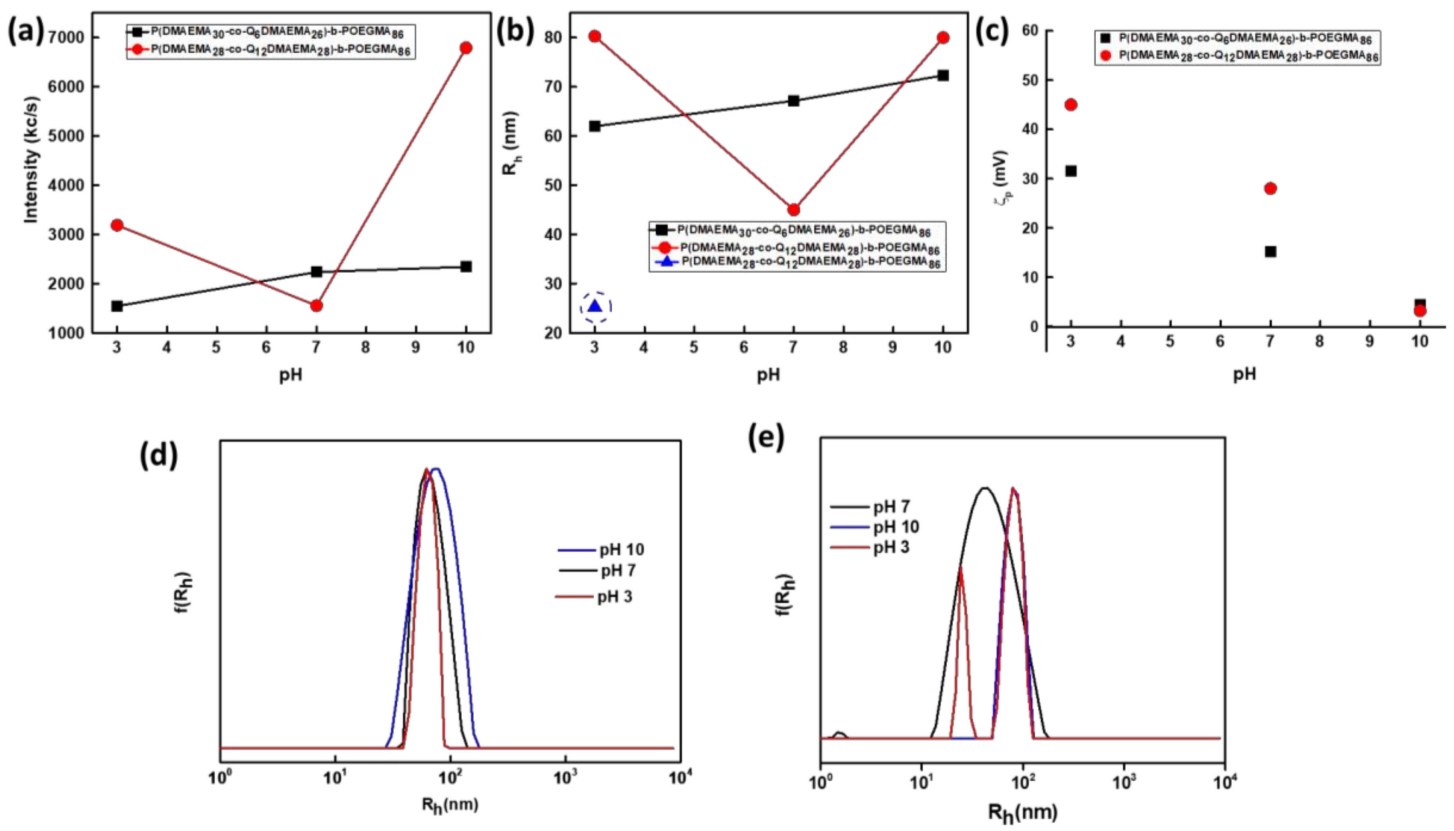
Dependence of (**a**) scattering intensity (Ι), (**b**) hydrodynamic radius (R_h_) and (**c**) surface charge (ζ_p_) for the P(DMAEMA_30_-co-Q_6_DMAEMA_26_)-*b*-POEGMA_86_ and P(DMAEMA_28_-co-Q_12_DMAEMA_28_)-*b*-POEGMA_86_ aqueous solutions on pH-variations and size distributions from CONTIN analysis obtained at 90^o^, at pH 7, 3 and 10 for (**d**) P(DMAEMA_30_-co-Q_6_DMAEMA_26_)-*b*-POEGMA_86_ and (**e**) P(DMAEMA_28_-co-Q_12_DMAEMA_28_)-*b*-POEGMA_86._ The preparation protocol via molecular dissolution in organic solvent was utilized and the concentration of the final polymer solution was 1 × 10^−3^ g/mL.

Response to pH changes is observed in both cases, however, the two diblocks exhibit different behavior, due to the fact that there are a longer alkyl chains present in the 2Q_12_ diblock. The self-assembly of the 2Q_12_ diblock at pH = 7, leads to the formation of aggregates of lower mass (scattering intensity is lower) and higher size (R_h_ =44 ± 2.2 nm) than 2Q_6_ (R_h_ = 66 ± 3.3 nm), indicating that fewer polymeric chains aggregate towards formation of nanoparticles. This is supported by the lower scattering intensity that sample 2Q_12_ exhibits at pH = 7, compared to 2Q_6_ sample and the obtained aggregation numbers (N_agg_ of 2Q_12_ = 79 (±4) and of 2Q_6_ = 85 (±4.3)). The incorporation of longer alkyl chains increases the hydrophobic character of the diblock chains and thus fewer molecules are required for the formation of stable nanoparticles. The pH-response of diblock 2Q_6_ is in agreement with the anticipated one. The protonation of the amino groups of the amino groups of DMAEMA that occurs at pH = 3 results in partial disaggregation of the originally formed nanostructures. The latter conclusion is based on the decrease in the scattering intensity (at pH = 3: I = 1550 kc/s (±78), at pH = 7: I = 2245 kc/s (±112)), that is accompanied with a decrease in the aggregation number (N_agg_ = 75). Even though, no significant change was monitored concerning the hydrodynamic radius (R_h_ = 76 nm (±3.8) at pH 3) indicating that the disaggregation results in “swelling” of the nanostructures. A slight increase in the scattering intensity occurs at pH = 10 (I = 2450 ± 123 kc/s), indicating further aggregation that is also supported by the increase of the aggregation number (N_agg_ = 102 ± 5.1). Similarly with the pH = 3 case, no major difference was monitored in the hydrodynamic radius, denoting that further aggregation results in more compact nanoparticles probably with less water molecules inside. Moreover, the pH response of the 2Q_6_ diblock is also confirmed from the size distribution. All three peaks are relatively narrow and symmetrical. The monomodal peaks proved once more the existence of one population at each case. From the position of each peak, it can be concluded that at pH = 10, nanostructures of slightly higher mass are present than the ones at pH 7 and 3, due to further aggregation that the deprotonation of the amino groups of the DMAEMA units induces. Moreover, the smaller nanoassemblies are observed at pH = 3 due to the disaggregation of original nanoparticles as a result of protonation of the non-modified amino groups. On the contrary, the response of the 2Q_12_ diblock at pH variations is unexpected. When the solution pH decreases to 3, further aggregation and formation of particles of larger mass occurs due to the presence of longer alkyl chains. The latter annotation is supported by the abrupt increase in scattering intensity (I = 3200 ± 160 kc/s), while the size of the nanoparticles almost doubles (R_h_ = 80 ± 4 nm) and a second population of nanoparticles appears (R_h_ = 25 ± 1.3 nm). The appearance of two distinct populations is also demonstrated at the size distribution from CONTIN at pH 3 (Figure 6e). At pH = 10, the major increase in the scattering intensity (I = 6800 ± 340 kc/s at pH = 10, while I = 1500 ± 75 kc/s at pH = 7), followed by the increase in size (R_h_ = 85 ± 4.3 nm) and aggregation number (N_agg_ = 120 ± 6) leads to the conclusion that further aggregation occurs at this pH. The explanation of this behavior at pH = 10 has been stated in similar cases. All the physicochemical data are presented in Table 4.

The R_g_/R_h0_ values obtained after extrapolation at zero angle fluctuate from 0.6 to 0.8, indicating the formation of compact nanoaggregates of spherical shape. 2Q_12_ diblock aggregates exhibit higher surface charge than 2Q_6_, at all pHs examined. Apparently, more cationic charges are located on the particle surface. In both cases, the higher values are recorded at pH 3 while the lower at pH 10 (in comparison with ones at pH 7) as expected. It should be also added that even though the lower ζ-potential values are encountered at pH = 10 they are positive as a consequence of the increased number of cations present along the PDMAEMA block due to the higher quaternization degree.

**Scheme 2:**
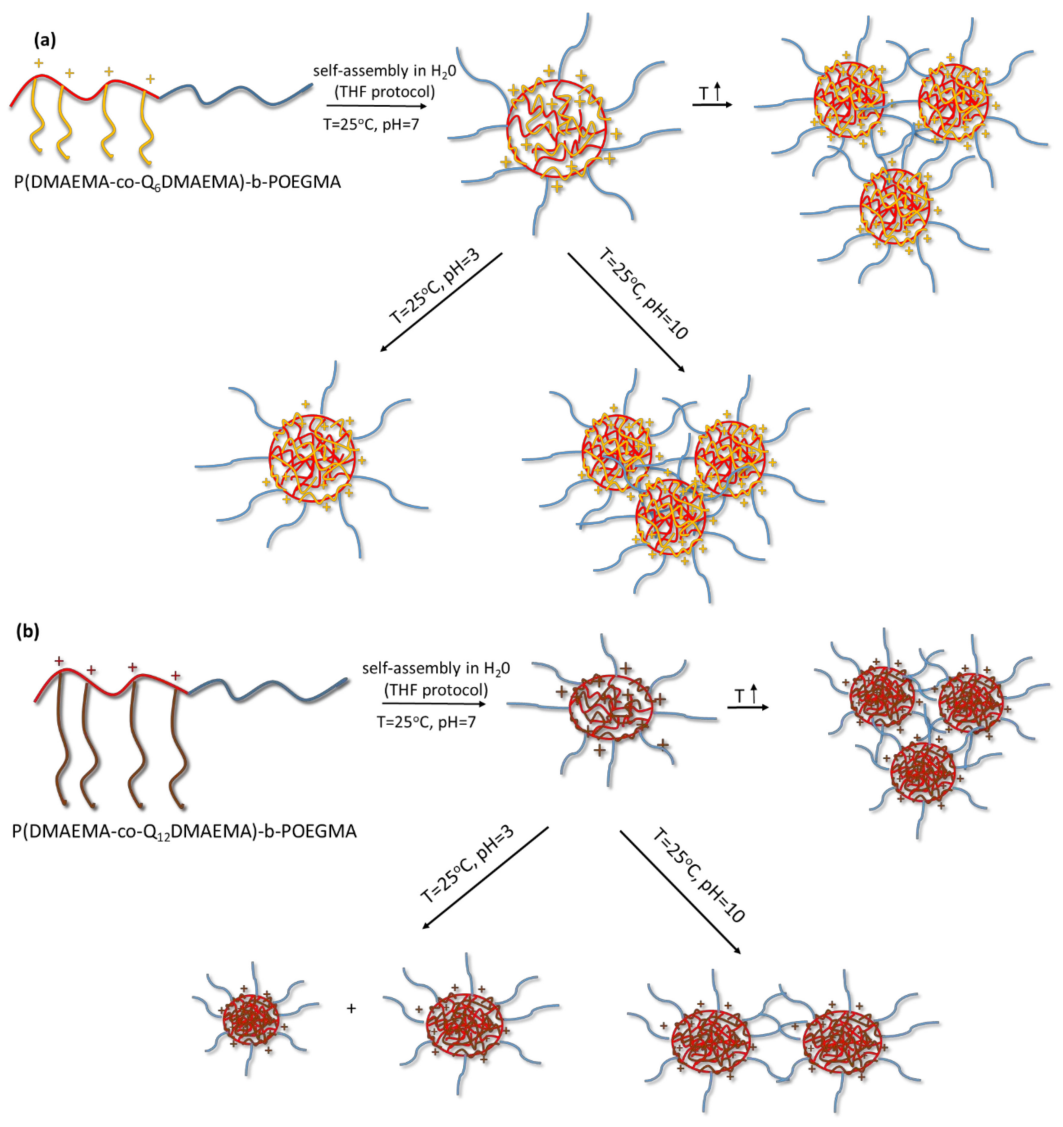
Graphical illustration of the pH and temperature response of the aqueous solutions of (**a**) P(DMAEMA-co-Q_6_DMAEMA)-*b*-POEGMA and (**b**) P(DMAEMA-co-Q_12_DMAEMA)-*b*-POEGMA diblocks_,_ when the preparation protocol included the initial molecular dissolution of the copolymers in THF.

At this point, it is worth mentioning that judging from the polydispersity indexes of the solutions prepared by using THF, they are more homogenous than the solutions prepared from direct copolymer dissolution. Generally, the dimensions of the nanoassemblies from the organic solvent protocol are lower. The above-mentioned solutions were stable for more than 15 days (as confirmed by DLS), while the ones prepared by direct dissolution in aqueous media showed some evidence of precipitation after 3 days from the day of their preparation. A graphical illustration of the self-assembly behavior of the random block copolymers is shown in Scheme 2.

#### 3.2.2. Temperature-Dependence of the Self-Assembly Behavior of the P(DMAEMA-co-Q_6_/_12_DMAEMA)-*b*-POEGMA Random Diblock Copolymers

The temperature response of the random diblock copolymers was investigated, in the case of the aqueous solutions prepared by the organic solvent protocol at 1 × 10^−3^ g/mL concentration. 

##### Temperature Responsiveness of P(DMAEMA_30_-co-Q_6_DMAEMA_26_)-*b*-POEGMA_86_ and P(DMAEMA_28_-co-Q_12_DMAEMA_28_)-*b*-POEGMA_86_

The partially quaternized samples, obtained from the first precursor diblock copolymer, with nominal quaternization degree ca. 20% did not show significant thermoresponsive behavior. The observation is attributed to the surrounding of the thermoresponsive DMAEMA segments by the hydrophobic side chains that make difficult the phase transition of DMAEMA to less solvated/ dehydrated state (see Table S2).

**Figure 7:**
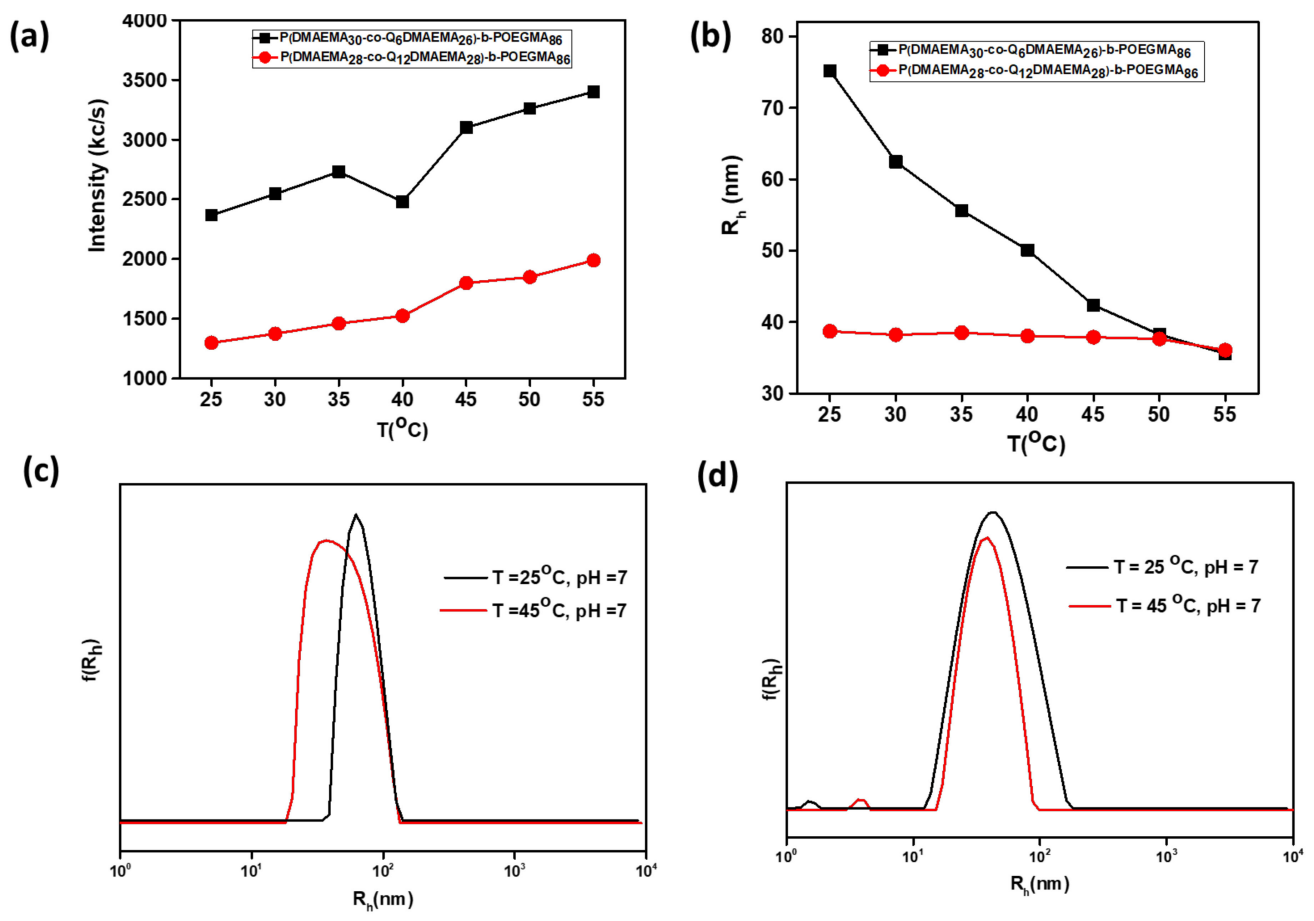
Dependence of (**a**) scattering intensity, (**b**) hydrodynamic radius for the P(DMAEMA_30_-co-Q_6_DMAEMA_26_)-*b*-POEGMA_86_ and P(DMAEMA_28_-co-Q_12_DMAEMA_28_)-*b*-POEGMA_86_ diblocks on temperature and size distributions from CONTIN obtained at 90° measurement angle, at T = 25 °C, pH = 7 and at T = 45 °C (for (**c**) P(DMAEMA_30_-co-Q_6_DMAEMA_26_)-*b*-POEGMA_86_ and (**d**) P(DMAEMA_28_-co-Q_12_DMAEMA_28_)-*b*-POEGMA_86_ solutions.

Observations of the scattering intensity, of both samples as a function of temperature from 25 °C to 55 °C, which indicates alterations in the mass of the resulted nanostructures, confirmed the secondary aggregation as temperature increases (Figure 7a). Specifically, for both diblocks the transition temperature to a more hydrophobic/ dehydrated state is above 40 °C. However, the two samples do not follow the same tendency regarding the size of the formed aggregates. The size of the 2Q_6_ aggregates decreases as temperature increases, probably due to the destruction of the hydrogen bonds between the DMAEMA segments and water molecules and thus intermolecular polymer interactions dominate. On the other hand, the size of the 2Q_12_ aggregates versus temperature appears rather constant (Figure 7b). A single population of nanoparticles, exists within the examined temperature range for both diblock solutions. This is confirmed by the size distributions from CONTIN, at T = 25 °C and 45 °C, where a monomodal peak appears (Figure 7c,d). All investigated solutions show high size homogeneity (PDI ≤ 0.2) within the examined temperature range.

##### Temperature Responsiveness of P(DMAEMA_22_-co-Q_6_DMAEMA_20_)-*b*-POEGMA_12_ and P(DMAEMA_21_-co-Q_12_DMAEMA_21_)-*b*-POEGMA_12_ Diblocks

**Figure 8:**
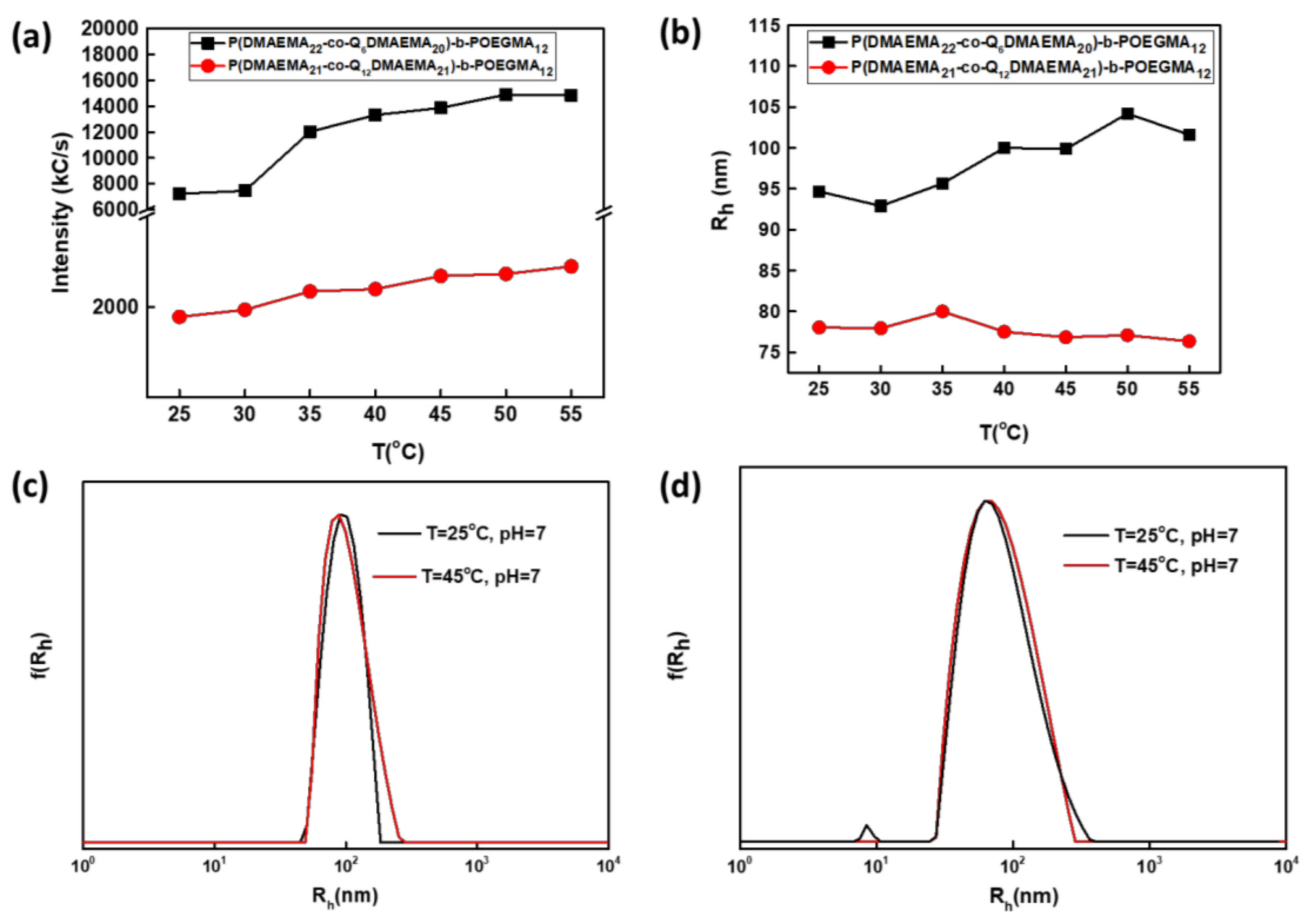
Dependence of (**a**) scattering intensity, (**b**) hydrodynamic radius for the P(DMAEMA_22_-co-Q_6_DMAEMA_20_)-*b*-POEGMA_12_ and P(DMAEMA_21_-co-Q_12_DMAEMA_21_)-*b*-POEGMA_12_ diblock solutions on temperature increase and size distribution from CONTIN obtained at 90^o^ measurement angle, at T = 25 °C, pH = 7) and at T = 45 °C for (**c**) P(DMAEMA_22_-co-Q_6_DMAEMA_21_)-*b*-POEGMA_12_ and (**d**) P(DMAEMA_21_-co-Q_12_DMAEMA_28_)-*b*-POEGMA_12_.

Investigations over the temperature response of the derivatives from the PDMAEMA_44_-*b*-POEGMA_12_ diblock precursor were conducted only in the case the nominal quaternization degree was 50% (Figure 8), because it is anticipated that the amplified hydrophobic character compared with the random copolymers that contain less hydrophobic chains, would lead to immediate temperature response.

The aforementioned hypothesis is confirmed in the case of diblock 4Q_6_, taking into account the dependence of the scattering intensity as temperature rises. The transition to a more hydrophobic/dehydrated state occurs at temperatures above 30 °C. The existence of more hydrophobic chains results in the transition to a less solvated state at a decreased temperature compared to sample 2Q_6._ It is worth mentioning that this comparison is qualitative even though both samples bear the same hydrophobic component (alkyl chain of six carbon atoms), but they are derived from diblocks that differ in PDMAEMA and POEGMA content. Subsequently, it is rather expected to behave differently towards temperature changes. The increase in the scattering intensity is accompanied with a small increase in hydrodynamic radius as secondary aggregation driven by the temperature rise results in nanoaggregates of both higher mass density and dimensions. The secondary aggregation is supported by the increase in the aggregation number from 25 °C to 45 °C. The monomodal hydrodynamic size distribution depicted in the size distribution graphs at both temperatures indicates the existence of only one population of aggregates, prompting to the conclusion that the aggregation tendency as temperature rises is similar. The weak thermoresponsive behavior is assigned to the large hydrophobic contribution of alkyl chains of twelve carbon atoms that surround the DMAEMA segments and do not permit further intramolecular interactions between the DMAEMA parts along the polymeric chain.

R_g_/R_h0_ ratios for the 4Q_6_ sample obtained at 25 °C and 45 °C, indicate the formation of compact spherical vesicles (Table 5). The high ζ-potential values are in complete agreement with the large number of cations attached to QDMAEMA segments, some of which are presumably oriented towards the surface of the particles. Increase in temperature causes conformational alterations. Therefore, more cations compared with T = 25 °C, are located on the surface of the particles and the value of the surface charge almost doubles. The non-thermoresponsive behavior of the 4Q_12_ is also verified by ELS measurements, as the surface charge remains essentially the same at both recorded temperatures. The higher value of the 4Q_12_ aggregates compared to 4Q_6_ ones may correspond to different inner morphology of the particles (Table 6).

### 3.3. Encapsulation and Release Studies of Indomethacin

One of the most interesting and studied applications of the amphiphilic block copolymers is the encapsulation of hydrophobic drugs. Random diblock copolymers were not so appealing for encapsulating hydrophobic drugs, before due to the inconsistency of the components along the polymeric chain. In the case of P(DMAEMA-co-Q_6/12_DMAEMA)-*b*-POEGMA block copolymers, the presence of long alkyl side chains enhances the hydrophobic character of the polymeric system and the inner region of aggregates formed in aqueous media should be structured by the random block where DMAEMA and QDMAEMA segments are segregated in a “scrambled egg” fashion. Subsequently, the inner aggregate region includes hydrophobic nano-domains formed by the aggregation of the long alkyl side chains, which can be used for the accommodation of hydrophobic drugs. Along with the rather biocompatible nature of the components of the block copolymers under study and their stimuli response, the random block copolymers are expected to be suitable nanomaterials for the encapsulation and delivery of the model hydrophobic drug indomethacin. From the eight random diblock copolymers synthesized, four were chosen for the encapsulation studies involving indomethacin, namely, the ones of nominal 50% quaternization degree due to their amplified hydrophobic character. The weight ratios (theoretical encapsulation degrees) of IND, relative to the entire random diblock copolymer was 10% and 20%wt. For brevity, the mixed samples would be referred as P(DMAEMA-co-Q_6/12_DMAEMA)-*b*-POEGMA/IND10% and P(DMAEMA-co-Q_6/12_DMAEMA)-*b*-POEGMA/IND20% respectively, according to the length of the alkyl chain and the weight composition of each mixture. DLS and SLS techniques were implemented to determine the mass, morphology and size of mixed nanoaggregates, and UV-Vis spectrophotometry for the assessment of the drug-loading efficiency. The obtained results are presented in Table 7.

**Figure 9:**
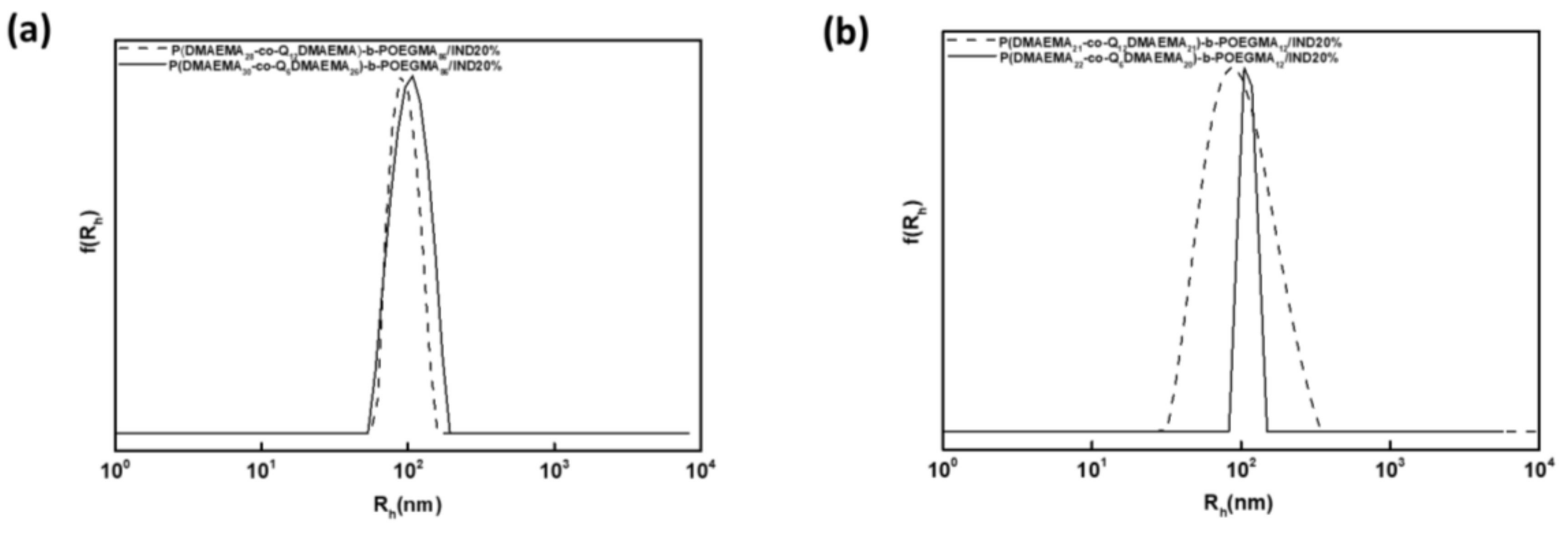
Size distributions from CONTIN for the mixed nanoassemblies resulting by the combination of IND at 20% and (**a**) partially quaternized PDMAEMA_56_-*b*-POEGMA_86_ with iodohexane and iodododecane and (**b**) partially quaternized PDMAEMA_44_-*b*-POEGMA_12_ with iodohexane and iodododecane.

An important observation, however is that the size of the mixed nanoparticles are larger than that of unloaded ones, indicating that the incorporation of IND into the inner hydrophobic domains contributes to the formation of larger polymer/drug aggregates. Moreover, the high scattered intensity measured is attributed to the formation of mixed nanoaggregates of high mass. The drug loading efficiency results are satisfactory although optimization of the encapsulation process is of the essence before stepping into real drug-delivery applications. Higher drug loading is obtained when higher initial drug quantity was used. From this observation the case of P(DMAEMA_22_-co-Q_6_DMAEMA_20_)-*b*-POEGMA_12_/IND20% is excluded probably due to structural features of the formed aggregates that do not favour the encapsulation of IND. In addition, a comparison on the drug loading efficiency should be performed according to the used quaternization agent along with the precursor diblock copolymer characteristics. When the PDMAEMA_56_-*b*-POEGMA_86_ copolymer was partially quaternized with iodohexane, higher drug loading (compared with the theoretical encapsulation values) were determined, denoting that the iodohexane functionalized diblock functions as a better encapsulation agent. On the other hand, when the PDMAEMA_44_-*b*-POEGMA_12_ diblock was partially quaternized with iodododecane, higher drug loading was obtained, demonstrating that the iodododecane acts as more appropriate encapsulation agent. It may be concluded that molecular characteristics of the initial diblocks and nature of quaternization agent play a significant role in random block copolymer/IND interactions and drug encapsulation. The aforementioned conclusion is also supported by the size distributions (Figure 9) that demonstrate higher sizes of the mixed nanoassemblies when the PDMAEMA_56_-*b*-POEGMA_86_ was quaternized with iodohexane and the PDMAEMA_44_-*b*-POEGMA_12_ was quaternized with iodododecane, for the same nominal quaternization degree. A possible explanation of the abovementioned observation is the encapsulation of higher drug quantity in these cases. 

In all cases monodisperse size distributions appear, confirming that one population of particles are dispersed in the aqueous media, which is desirable for drug delivery applications. Moreover, the higher POEGMA composition seems to contribute in the encapsulation of larger amount of IND due to better colloidal stability and increased solvation of the drug loaded aggregates.

**Figure 10:**
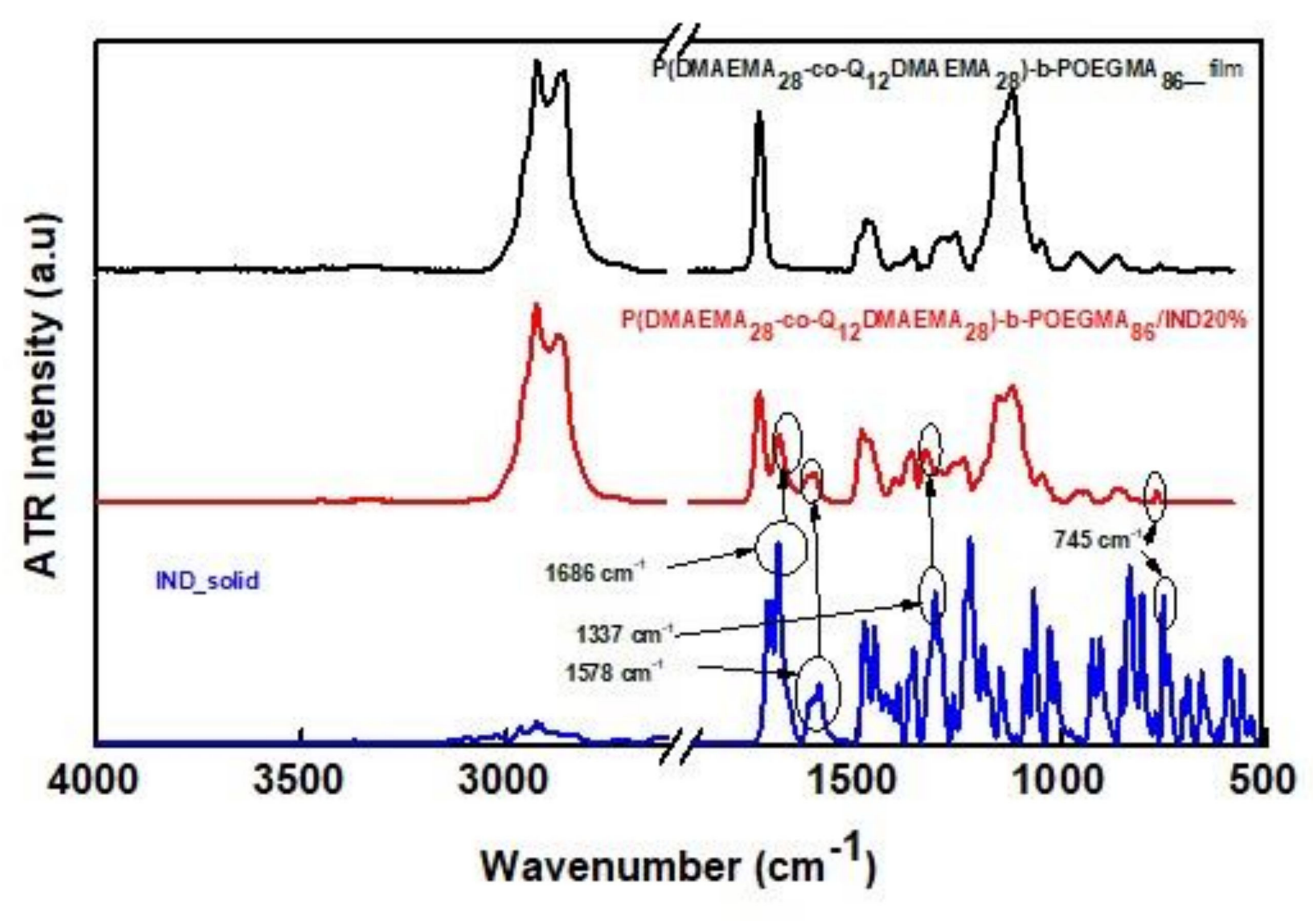
Comparative ATR-FTIR spectra of the mixed P(DMAEMA_2__8_-co-Q_12_DMAEMA_2__8_)-*b*-POEGMA_86_/IND20% aggregates the P(DMAEMA_28_-co-Q_12_DMAEMA_28_)-*b*-POEGMA_86_ empty aggregates and IND in the solid state.Note: The ATR-FTIR spectra of aqueous solutions of random diblock copolymer and mixed nanoassemblies of random diblock copolymer with IND emanated after the evaporation of water by using nitrogen, so that the water absorption peaks would not intervene with the characteristic absorption peaks that would reveal the interactions between IND and the random diblock copolymer.

The interactions existing between the encapsulated IND and the random diblock copolymers in the case of P(DMAEMA_28_-co-Q_12_DMAEMA_28_)-*b*-POEGMA_86_/IND20% and P(DMAEMA_21_-co-Q_12_DMAEMA_21_)-*b*-POEGMA_12_/IND20% assemblies were studied by ATR-FTIR spectroscopy (Figure 10).

The spectra of the mixed P(DMAEMA_21_-co-Q_12_DMAEMA_21_)-*b*-POEGMA_12_/IND20% aggregates with the P(DMAEMA_21_-co-Q_12_DMAEMA_21_)-*b*-POEGMA_12_ empty aggregates are presented in the SI (Figure S6). Those two samples were selected to be investigated by ATR-FTIR because they present the higher loading efficiency and the physical interactions between vectors and IND would be stronger and more easily identifible.

Regarding the ATR-FTIR spectrum of solid indomethacin, the characteristic absorption band at 1686  cm^−1^ is assigned to amide I group and specifically to the stretching vibration of the C=O group, the one at 1578  cm^−1^ is assigned to C=C stretching vibration of the aromatic rings. Moreover, the absorption peak at 1337 cm^−1^ is assigned to the asymmetric stretching of the C-O group and the one at 745 cm^−1^ corresponds to the C-Cl stretching vibration [65,66]. Regarding the spectrum of the P(DMAEMA_28_-co-Q_12_DMAEMA_28_)-*b*-POEGMA_86_/IND20% mixed aqueous solution, the characteristic IND absorption peaks appear, indicating the strong physical interactions between P(DMAEMA_28_-co-Q_12_DMAEMA_28_)-*b*-POEGMA_86_ diblock and the encapsulated IND. The absence of these characteristic absorption peaks in the ATR-FTIR spectrum of the P(DMAEMA_21_-co-Q_12_DMAEMA_21_)-*b*-POEGMA_12_ diblock (black line) confirms the encapsulation of IND into the polymeric nanoaggregates.

**Figure 11:**
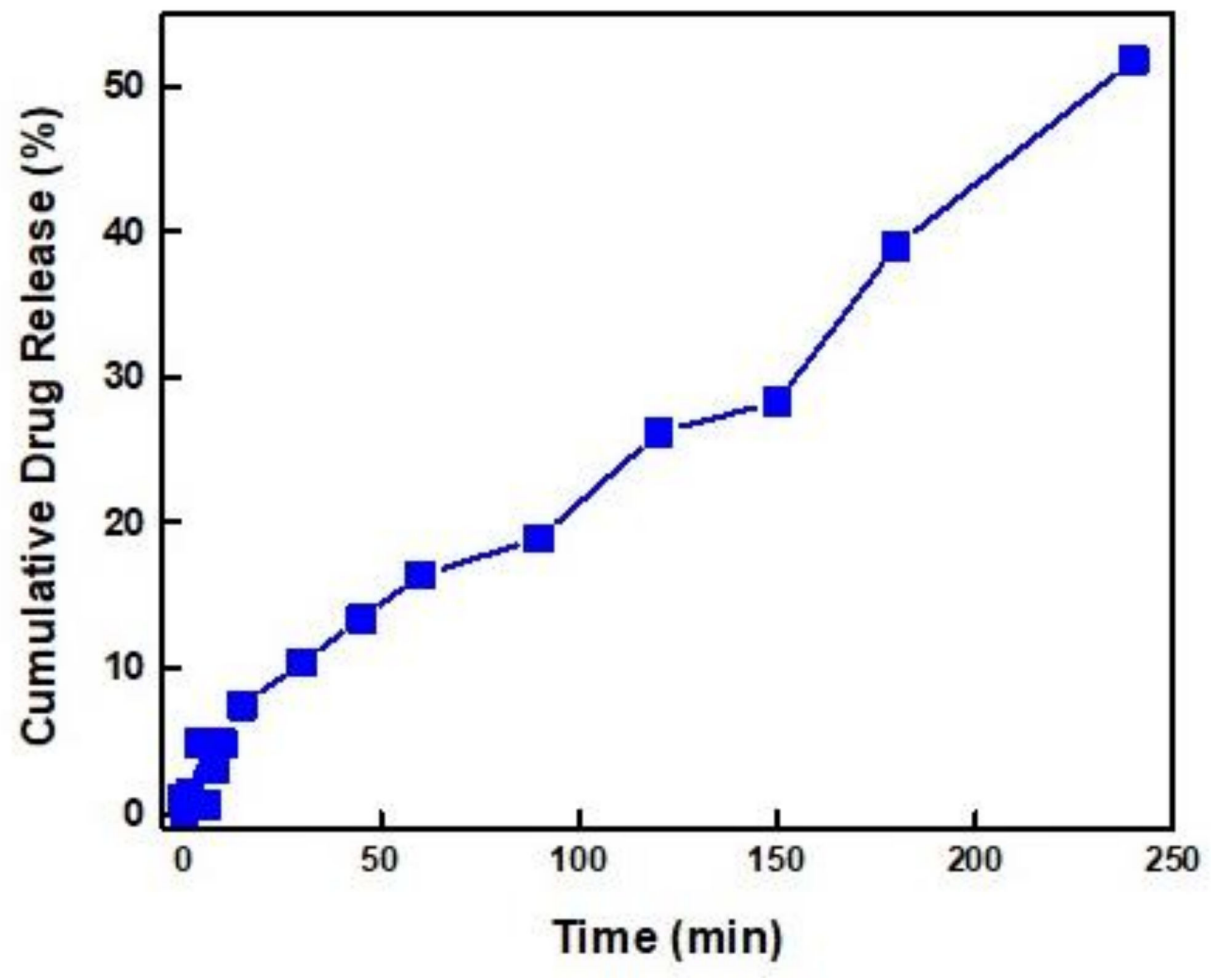
Release of IND from P(DMAEMA_28_-co-Q_12_DMAEMA_28_)-*b*-POEGMA_86_ containing nanoaggregates under sonication.

The release of IND from P(DMAEMA_28_-co-Q_12_DMAEMA_28_)-*b*-POEGMA_86_ containing nanoaggregates under sonication, is presented in Figure 11.

Drug release studies were performed at the P(DMAEMA_28_-co-Q_12_DMAEMA_28_)-*b*-POEGMA_86_/IND20% sample. The specific nanosystem was chosen due to the high drug loading efficiency, the high aggregate mass (determined by the high scattering intensity value) and the good colloidal stability over time (more than 15 days) that this sample exhibited due to the good solubility characteristics that the high POEGMA content provide. Initially, release studies were performed at sink conditions at room temperature. No appreciable release of IND was observed for 12 h. The release studies were performed using an ultrasonic bath in order to aid the release of IND. The absorbance of each sample was recorded by UV-Vis spectrophotometer in pre-defined time intervals. The highest rate of IND release was monitored after 4 h since the beginning of the ultrasonication process and determined to be almost 50%. It was also noticed that the release of indomethacin starts almost at the same time with the application of ultrasonic radiation and it is constantly increasing throughout the release experiment, which lasted for four hours, without reaching a plateau. The relatively high IND release implies that a great portion of the indomethacin is not tightly bound to the polymeric nanoaggregates and it is more easily released. However, the intensity of the formed interactions between drug and polymer is adequate in order to retain the indomethacin encapsulated in the polymeric nanoaggregates in the absence of sonication, which in turn allows for an externally triggered and controlled release of the drug from the particular random block copolymer nanocarriers.

## 4. Conclusions

Novel random diblock copolymers were prepared via partial hydrophobic chemical modification of PDMAEMA-*b*-POEGMA precursor double hydrophilic diblock copolymers, which were synthesized by RAFT polymerization. The quaternization agents utilized were iodohexane and iodododecane. The molecular characterization methods confirmed the synthesis of relatively well-defined block copolymers. The experimental quaternization degrees found by ^1^H-NMR were in accordance with the theoretical values calculated by using the molar ratio of the alkyl halide to the DMAEMA monomeric units during chemical functionalization. Light scattering techniques showed the formation of nanoaggregates of high mass along with relatively low size in aqueous media. The self-assembly of the random diblock copolymers that bear alkyl chains of twelve carbon atoms led to formation of nanoaggregates of lower mass due to the enhanced hydrophobic character of the polymeric chains. Unimolecular dissolved copolymer chains were also detected in some cases. The ability of the hydrophobically modified diblock copolymers to respond to pH and temperature changes remained, but it greatly depended on the quaternization degree, nature of the alkyl side chain and preparation protocol. Generally, nanoaggregates of the aqueous solutions prepared by implementing the organic solvent protocol (using THF) were better defined judging from their rather low polydispersity indexes, better colloidal stability over time and exhibited significant response to pH and temperature. The imparted hydrophobic character led to significantly lower transition temperature than the reported LCST of PDMAEMA homopolymer. In the case of P(DMAEMA_30_-co-Q_12_DMAEMA_26_)-*b*-POEGMA_86_ copolymer, when the preparation protocol included the use of organic solvent the R_g_/R_h0_ parameter acquired values above 1, indicating the possible formation of spherical vesicles. ELS investigations showed rather high positive surface charge assigned to the morphology of the nanoaggregates where the hydrophobic chains obtained such configuration that allows the positive charges to be located on the surface. The increase in the quaternization degree was accompanied with an increase in the surface charge. Subsequently, due to the increased hydrophobic character of the diblocks encapsulation studies of the inflammatory, hydrophobic drug indomethacin, was achieved. The resulted IND loaded nanoparticles were larger in size due to the hydrophobic nature of indomethacin. Drug loading efficiency studies showed the successful encapsulation of indomethacin. Indomethacin release experiments under sonication demonstrated the successful release of a high proportion of the drug. Overall, the side group nature and particular molecular characteristics of the hydrophobically modified P(DMAEMA-co-Q_6/12_DMAEMA)-*b*-POEGMA random diblock copolymers, along with their tunable pH and temperature response makes them another novel example of amphiphilic copolymers with controlled and unprecedented self-assembly characteristics and aggregate structure appealing for utilization in bionanotechnological applications, including drug, gene and protein delivery. 

## Figures and Tables

**Table 1:** Molecular characteristics of partially chemically modified P(DMAEMA-co-Q_6/12_DMAEMA)-*b*-POEGMA random diblock copolymers and their precursors.

Sample	M_W_^a^ (×10^4^) (g/mol)	M_W_/Mn ^a^	M_wQDMAEMA_^a^ (×10^4^) (g/mol)	%wt PDMAEMA ^b^	%wt POEGMA ^b^	%wt QDMAEMA ^a^	Quaternization Degree (%) ^b^
PDMAEMA_56_	0.9	1.13	-	100	-	-	-
PDMAEMA_56_-*b*-POEGMA_86_	4.95	1.4	-	16	82	-	-
P(DMAEMA_45_-co-Q_6_DMAEMA_11_)-*b*-POEGMA_86_	5.2 ^c^	-	0.4	14	78	8	19
P(DMAEMA_30_-co-Q_6_DMAEMA_26_)-*b*-POEGMA_86_	5.5 ^c^	-	1.0	9	74	17	46
P(DMAEMA_44_-co-Q_12_DMAEMA_12_)-*b*-POEGMA_86_	5.3 ^c^	-	0.55	13	77	10	20
P(DMAEMA_28_-co-Q_12_DMAEMA_28_)-*b*-POEGMA_86_	5.8 ^c^	-	1.3	7	71	22	50
PDMAEMA_42_	0.65	1.15	-	100	-	-	-
PDMAEMA_42_-*b*-POEGMA_1__2_	1.2	1.4	-	55	45	-	-
P(DMAEMA_34_-co-Q_6_DMAEMA_8_)-*b*-POEGMA_12_	1.4 ^c^	-	0.3	39	39	22	19
P(DMAEMA_22_-co-Q_6_DMAEMA_20_)-*b*-POEGMA_12_	1.6 ^c^	-	0.75	21	33	46	48
P(DMAEMA_33_-co-Q_12_DMAEMA_9_)-*b*-POEGMA_12_	1.5 ^c^	-	0.4	36	36	28	20
P(DMAEMA_21_-co-Q_12_DMAEMA_21_)-*b*-POEGMA_12_	1.8 ^c^	-	1.0	18	29	53	50

^a^ Determined by SEC in THF, containing 5% trimethylamine in THF, ^b^ Determined by ^1^H-NMR, ^c^ Determined by SLS in THF. Note: Values of the weight fractions and the quaternization degrees were determined with standard deviations of ±1%.

**Table 2:** Physicochemical characteristics of P(DMAEMA_45_-co-Q_6_DMAEMA_11_)-*b*-POEGMA_86_ and P(DMAEMA_44_-co-Q_12_DMAEMA_12_)-*b*-POEGMA_86_ diblock solutions resulting from the direct dissolution protocol at different pHs (c = 5 × 10^−3^ g/mL).

Direct Dissolution Preparation Protocol							
Sample	Quaternization Degree (%) ^a^	pH	R_h_^b,e^ (nm)	PDI ^b^	N_agg_^c,e^	R_g_/R_h0_^b,c^	ζ_p_^d,e^ (mV)
P(DMAEMA_45_-co-Q_6_DMAEMA_11_)-*b*-POEGMA_86_ (1Q_6_)	19	3	108	0.35	83	0.75	11
		7	99	0.32	55	0.65	8.3
		10	105	0.29	90	0.8	−1
P(DMAEMA_44_-co-Q_12_DMAEMA_12_)-*b*-POEGMA_86_ (1Q_12_)	20	3	5/150	0.44	37	-	12
		7	5/133	0.41	30	-	9.98
		10	6/107	0.45	23	-	3

^a^ Determined by ^1^H-NMR, ^b^ Determined by DLS at measuring angle 90°, ^c^ Determined by SLS, ^d^ Determined by ELS, ^e^ Values of R_h_, N_agg_ and ζ_p_ were determined with a standard deviation of ±5%. Note: The two different Rh values correspond to two different populations detected in the solution. The smaller one (fist value) is assigned to unimers, while the bigger one (second value) is assigned to aggregates.

**Table 3:** Physicochemical features of the P(DMAEMA_30_-co-Q_6_DMAEMA_26_)-*b*-POEGMA_86_ and P(DMAEMA_28_-co-Q_12_DMAEMA_28_)-*b*-POEGMA_86_, at direct dissolution protocol and solution concentration c = 5 × 10^−3^ g/mL, extracted from pH-response studies.

Direct Dissolution Preparation Protocol							
Sample	Quaternization Degree (%) ^a^	pH	R_h_^b,e^ (nm)	PDI ^b^	N_agg_^c,e^	R_g_/R_h0_^b,c^	ζ_p_^d,e^ (mV)
P(DMAEMA_30_-co-Q_6_DMAEMA_26_)-*b*-POEGMA_86_ (2Q_6_)	46	3	124	0.29	60	0.6	26
		7	206	0.4	65	0.7	12
		10	167	0.37	45	0.67	0.9
P(DMAEMA_28_-co-Q_12_DMAEMA_28_)-*b*-POEGMA_86_ (2Q_12_)	50	3	36	0.4	45	0.7	13
		7	69	0.45	110	0.8	3.2
		10	72	0.35	72	0.72	−0.27

^a^ Determined by ^1^H-NMR, ^b^ Determined by DLS at a measuring angle 90°, ^c^ Determined by SLS, ^d^ Determined by ELS, ^e^ Values of R_h_, N_agg_ and ζ_p_ were determined with a standard deviation of ± 5%.

**Table 4:** Physicochemical characteristics of the P(DMAEMA_30_-co-Q_6_DMAEMA_26_)-*b*-POEGMA_86_ and P(DMAEMA_28_-co-Q_12_DMAEMA_28_)-*b*-POEGMA_86_ diblocks at different pHs, from organic solvent protocol and solution concentration c = 1 × 10^−3^ g/mL.

Organic Solvent (THF) Preparation Protocol							
Sample	Quaternization Degree (%) ^a^	pH	R_h_^b,e^ (nm)	PDI ^b^	N_agg_^c,e^	R_g_/R_h0_^b,c^	ζ_p_^d,e^ (mV)
P(DMAEMA_30_-co-Q_6_DMAEMA_26_)-*b*-POEGMA_86_ (2Q_6_)	46	3	62	0.32	75	0.6	+32
		7	66	0.35	85	0.72	+15
		10	72	0.3	102	0.63	+3
P(DMAEMA_28_-co-Q_12_DMAEMA_28_)-*b*-POEGMA_86_ (2Q_12_)	50	3	25/80	0.33	83	-	+46
		7	44	0.38	79	0.65	+28
		10	85	0.35	150	0.8	+3.5

^a^ Determined by ^1^H-NMR, ^b^ Determined by DLS at measuring angle 90°, ^c^ Determined by SLS, ^d^ Determined by ELS, ^e^ Values of R_h_, N_agg_ and ζ_p_ were determined with a standard deviation of ±5%. Note: The two different R_h_ values correspond to two different populations detected in the solution. The smaller one (fist value) is assigned to smaller aggregates, while the bigger one (second value) is assigned to bigger aggregates.

**Table 5:** Physicochemical characteristics of the P(DMAEMA_30_-co-Q_6_DMAEMA_26_)-*b*-POEGMA_86_ and P(DMAEMA_28_-co-Q_12_DMAEMA_28_)-*b*-POEGMA_86_ diblocks at two temperatures in solutions prepared by the organic solvent protocol (c = 1 × 10^−3^ g/mL).

Sample	Quaternization Degree (%) ^a^	Τ (°C)	R_h_^b,e^ (nm)	PDI ^b^	N_agg_^c,e^	ζ_p_^d,e^(mV)
P(DMAEMA_30_-co-Q_12_DMAEMA_26_)-*b*-POEGMA_86_ (2Q_6_)	46	25	68	0.195	85	+15.2
		45	45	0.173	103	+19
P(DMAEMA_28_-co-Q_12_DMAEMA_28_)-*b*-POEGMA_86_ (2Q_12_)	50	25	45	0.20	79	+28
		45	38	0.192	91	+35

^a^ Determined by ^1^H-NMR, ^b^ Determined by DLS at measuring angle 90°, ^c^ Determined by SLS, ^d^ Determined by ELS, ^e^ Values of R_h_, N_agg_ and ζ_p_ were determined with a standard deviation of ±5%. Note: The two different Rh values correspond to two different populations detected in the solution. The smaller one (fist value) is assigned to unimers, while the bigger one (second value) is assigned to aggregates. The difference in aggregation numbers at T = 25 °C and 45 °C, also confirms the further aggregation phenomena that occur because of the increased hydrophobic character induced by the temperature increase. ΕLS investigations showed increase of the surface charge for both diblocks when temperature increases from 25 °C to 45 °C. The latter observation corresponds to morphological changes that arise as temperature increases, promoting a higher number of positive charges near the particle surface, also as a result of the increased number of chains per aggregate.

**Table 6:** Physicochemical characteristics of P(DMAEMA_22_-co-Q_6_DMAEMA_20_)-*b*-POEGMA_12_ and P(DMAEMA_21_-co-Q_12_DMAEMA_21_)-*b*-POEGMA_12_ diblock solutions prepared by the organic solvent protocol (c = 1 × 10^−3^ g/mL), at two different temperatures.

Sample	Quaternization degree (%) ^a^	Τ (°C)	R_h_^b^^,e^ (nm)	PD.I ^b^	N_agg_^c,e^	R_g_/R_h0_^b,c^	ζ_p_^d,e^ (mV)
P(DMAEMA_30_-co-Q_12_DMAEMA_26_)-*b*-POEGMA_86_ (4Q_6_)	48	25	95	0.128	145	0.99	+26.1
		45	100	0.126	279	0.9	+46.5
P(DMAEMA_28_-co-Q_12_DMAEMA_28_)-*b*-POEGMA_86_ (4Q_12_)	50	25	78	0.229	83	0.64	+54.3
		45	66	0.215	87	1.06	+58.4

^a^ Determined by ^1^H-NMR, ^b^ Determined by DLS at measuring angle 90°, ^c^ Determined by SLS, ^d^ Determined by ELS, ^e^ Values of R_h_, N_agg_ and ζ_p_ were determined with a standard deviation of ±5%.

**Table 7:** Physicochemical characteristics of the mixed random diblock copolymers with IND and the drug loading results of all the examined mixed nanoparticles.

Sample	Stoichiometry of Encapsulation ^a^ (% *w*/*w*)	Intensity ^b^^,^^f^ (kC/s)	R_h_^c,f^ (nm)	R_g_/R_h0_^b^	% Drug loading ^d,f^	ζp ^e,f^ (mV)
P(DMAEMA_30_-co-Q_6_DMAEMA_26_)-*b*-POEGMA_86_/IND10%	10	872	125.17	0.55	5.5	+21
P(DMAEMA_28_-co-Q_12_DMAEMA_28_)-*b*-POEGMA_86_/IND10%	10	2990	82.25	0.75	4	+11.5
P(DMAEMA_22_-co-Q_6_DMAEMA_20_)-*b*-POEGMA_12_/IND10%	10	1654	121.97	0.63	3	+45
P(DMAEMA_21_-co-Q_12_DMAEMA_21_)-*b*-POEGMA_12_/IND10%	10	1456	102.06	0.5	6	+44.6
P(DMAEMA_30_-co-Q_6_DMAEMA_26_)-*b*-POEGMA_86_/IND20%	20	2555	106.86	0.5	7.2	+28
P(DMAEMA_28_-co-Q_12_DMAEMA_28_)-*b*-POEGMA_86_/IND20%	20	32000	93.15	0.84	7	+23
P(DMAEMA_22_-co-Q_6_DMAEMA_20_)-*b*-POEGMA_12_/IND20%	20	2570	113.22	0.5	5.5	+52
P(DMAEMA_21_-co-Q_12_DMAEMA_21_)-*b*-POEGMA_12_/IND20%	20	1950	94.30	0.53	6.3	+50

^a^ Calculated according to the amount of the copolymer, used ^b^ Determined by DLS at measuring angle 90°, ^c^ Determined by SLS, ^d^ Determined by calculating the ratio of IND weight in the nanoparticles, based on UV-Vis absorption values, to the weight of the used IND, ^e^ Determined by ELS, ^f^ Values were determined with a standard deviation of ±5%.

## Data Availability

Data are available upon request.

## Supplementary Materials

The following are available online at https://www.mdpi.com/2073-4360/13/3/338/s1, Figure S1: SEC chromatograms of PDMAEMA_42_ (first block) and PDMAEMA_42_-*b*-POEGMA_12_ (final diblock copolymer), Figure S2: ^1^H-NMR spectrum for PDMAEMA_44_-*b*-POEGMA_12_ in CDCl_3_, Figure S3: Comparative ATR-FTIR spectra of (a) PDMAEMA_56_-*b*-POEGMA_86_ precursor diblock copolymer (red line) and (b) P(DMAEMA_45_-*b*-Q_6_DMAEMA_11_)-*b*-POEGMA_86_ resulted random diblock copolymer (blue line), Figure S4: Dependence of (a) scattering intensity (I), (b) hydrodynamic radius P(DMAEMA_34_-co-Q_6_DMAEMA_8_)-*b*-POEGMA_12_ and P(DMAEMA_33_-co-Q_12_DMAEMA_9_)-*b*-POEGMA_12_ aqueous solutions on pH-variations (THF protocol), Figure S5: Dependence of (a) scattering intensity (I), (b) hydrodynamic radius P(DMAEMA_22_-co-Q_6_DMAEMA_20_)-*b*-POEGMA_12_ and P(DMAEMA_21_-co-Q_12_DMAEMA_21_)-*b*-POEGMA_12_ aqueous solutions on pH-variations (THF protocol), Figure S6: Comparative ATR-FTIR spectra of the mixed P(DMAEMA_21_-co-Q_12_DMAEMA_21_)-*b*-POEGMA_12_/IND20% (black line), the P(DMAEMA_21_-co-Q_12_DMAEMA_21_)-*b*-POEGMA_12_ empty vector (red line) and IND at solid state (blue line), Table S1: Scattering intensity and R_h_ results for the random diblock copolymers, obtained by the partial quaternization of the PDMAEMA_56_-*b*-POEGMA_86_ precursor, Table S2: Scattering intensity and R_h_ results for the 1Q_6_ and 1Q_12_ samples at T = 25 °C and T = 45 °C.
