# Supplementary material for: Effects of Hydrophobic Modifications on the Solution Self-Assembly of P(DMAEMA-co-QDMAEMA)-b-POEGMA Random Diblock Copolymers
Source: Polymers (Basel). 2021 Jan 21;13(3):338. doi: 10.3390/polym13030338 (PMC7866081; doi:10.3390/polym13030338)
Supplement: Supplementary file 1 [file polymers-13-00338-s001.pdf]

polymers

MDPI

Supplementary Materials

Effects of Hydrophobic Modifications on the Solution Self-Assembly of P(DMAEMA-co-QDMAEMA)-b-POEGMA Random Diblock Copolymers

Martha Kafetzi and Stergios Pispas*

Theoretical and Physical Chemistry Institute, National Hellenic Research Foundation, 48 Vassileos Constantinou Avenue, 11635 Athens, Greece; mkafetzi91@gmail.com
* Correspondence: pispas@eie.gr; Tel.: +30-210-727-3824

1. Additional Experimental details

Synthesis of PDMAEMA-b-POEGMA Diblock Copolymers

The diblock copolymers were synthesized by utilizing consecutive RAFT polymerization technique as reported elsewhere [1]. Briefly, in a 25 mL round bottom flask , equipped with a magnetic stirrer, 2-(dimethylamino)ethyl methacrylate (3 g, 19.08 mmol), 4-cyano-4-[(dodecylsulfanylthiocarbonyl)sulfanyl] pentanoic acid (CPD)(0.173 g, 0.43 mmol), 2,2-azobis(isobutyronitrile) (AIBN)(0.007 g, 0.043 mmol) and 12 mL 1,4-dioxane were introduced. The flask was sealed with a rubber septum. The reaction solution was degassed by high purity nitrogen gas bubbling for 20 minutes and was submerged in an oil bath at 65°C for 18h. Afterwards, the homopolymerization was terminated by cooling the mixture at -20°C and exposing it to air. The homopolymer was precipitated in excess n-hexane twice, after re-dissolution in THF in order to remove unreacted monomer. The product was collected after drying in a vacuum oven at 25°C for 48h (yield 87%). Subsequently, the PDMAEMA block was used as the macro-CTA agent to polymerize OEGMA and finally produce PDMAEMA-b-POEGMA diblock copolymer. The synthesis process that was followed in order to produce PDMAEMA-b-POEGMA diblock copolymers is described below. PDMAEMA macro-CTA (0.5 g, 0.06 mmol), (oligo ethylene glycol)methacrylate (2.5 g, 5.21 mmol), AIBN (0.002 g, 0.012 mmol) and 12 mL 1,4-dioxane were added in a 25 mL round bottom flask. The reaction mixture was degassed by high purity nitrogen gas bubbling for 20 minutes and then placed in an oil bath at 70°C for 24h. The polymerization was quenched by adjusting the temperature of the mixture at -20°C and exposure to air. Subsequently, the diblock copolymer was precipitated in excess n-hexane for removal of the unreacted OEGMA, and then left for 48h inside a vacuum oven at 25°C to dry (yield 85%). By following the above mentioned procedure, two PDMAEMA homopolymers were prepared. They differ in terms of molecular weight (M_w) and polydispersity index (M_w/M_n). Each PDMAEMA homopolymer was utilized as the macro-CTA to produce one PDMAEMA-b-POEGMA diblock copolymer. The two diblock copolymers present different molecular weights (M_w), polydispersity index (M_w/M_n) and composition (Table 1). Size exclusion chromatography (SEC) was used to determine the molecular weight and the polydispersity index of the PDMAEMA homopolymers and the resulted PDMAEMA-b-POEGMA diblock copolymers. The chemical structure and composition of the diblock copolymers were confirmed by proton nuclear magnetic resonance spectroscopy (^{1}H-NMR).

Partial Quaternization of DMAEMA-based Diblock Copolymers using Iodohexane and Iodododecane as Quaternization Agents

Partial Chemical Modification via Quaternization Reaction of the Precursor PDMAEMA-b-POEGMA using Iodohexane as the Quaternization Agent

The PDMAEMA block of the PDMAEMA-b-POEGMA precursor diblock copolymers was partially quaternized by iodohexane ($C_6H_{13}I$) to provide P(DMAEMA-co-Q_6DMAEMA)-b-POEGMA diblock copolymers with a cationic random amphiphilic block. The nominal quaternization degrees were 20 and 50% and referred to the % molar ratio of iodohexane to DMAEMA units. The process of the modification is described in the following. 0.25 g of PDMAEMA-*b*-POEGMA diblock copolymer were dissolved in 12.5 mL of THF (2% w/v) and 0.0103 g (0.48 mmol) $C_6H_{12}I$ ($C_6H_{12}I$ moles / DMAEMA moles = 0.2) were added in a round bottom flask when the polymer was completely dissolved in THF. The reaction was carried out at room temperature under stirring for 48h. Afterwards, the mixture was precipitated in excess of n-hexane for removal of the unreacted iodohexane, and then left for 48h inside a vacuum oven at 25°C to dry. The same procedure was followed also in the case where the molar ratio of iodohexane to DMAEMA units was 50%. The quaternization degree was assessed by ^{1}H-NMR spectroscopy while ATR-FTIR spectroscopy was utilized in order to confirm the chemical structure of the partially chemically-modified copolymers.

Partial Chemical Modification via Quaternization Reaction of the Precursor PDMAEMA-b-POEGMA using Iodododecane as the Quaternization Agent

The PDMAEMA block of the PDMAEMA-b-POEGMA precursor diblock copolymers was partially quaternized by iodododecane ($C_{12}H_{25}I$) to provide P(DMAEMA-co-Q_{12}DMAEMA)-b-POEGMA diblock copolymers with a cationic random amphiphilic block. The nominal quaternization degrees were 20 and 50% and referred to the % molar ratio of iodododecane to DMAEMA units. The process of the modification is described in the following. 0.25 g of PDMAEMA-*b*-POEGMA diblock copolymer were dissolved in 12.5 mL of THF (2% w/v) and 0.0143 g $C_{12}H_{25}I$ ($C_{12}H_{25}I$ moles / PDMAEMA moles = 0.5) were added in a round bottom flask when the polymer was completely dissolved in THF. The reaction was carried out at room temperature under stirring for 48h. Afterwards, the mixture was precipitated in excess of n-hexane for removal of the unreacted iodododecane, and then left for 48h inside a vacuum oven at 25°C to dry. The same procedure was followed also in the case of 50% modification. The quaternization degree was determined by ^{1}H-NMR spectroscopy while ATR-FTIR spectroscopy was utilized in order to confirm the chemical structure of the modified copolymers. Below, the ^{1}H-NMR chemical shifts of the P(DMAEMA-co-Q_6DMAEMA)-b-POEGMA and P(DMAEMA-co-Q_{12}DMAEMA)-b-POEGMA are presented.

^{1}H-NMR spectral peaks of P(DMAEMA-co-Q_6DMAEMA)-b-POEGMA, 600 MHZ, D_2O-d1, δ): 4.52 (2H, - $COOCH_2$ $CH_2N^+((CH_3)_2(CH_2))$-) 4.22 (2H, -($COOCH_2$ CH_2N-), 3.74 (36H, -$(CH_2CH_2O)_9CH_3$-), 3.54 (2H, - $COOCH_2$ $CH_2N^+((CH_3)_2(CH_2))$-), 3.4 (3H, -$(CH_2CH_2O)_9CH_3$-), 3.35 (6H, - $COOCH_2$ $CH_2N^+((CH_3)_2(CH_2))$-), 2.85 (2H, - $COOCH_2$ $CH_2N^+((CH_3)_2(CH_2))$-) , 2.65 (2H, -($COOCH_2$ CH_2N-) , 2.42 (6H, -(6H, -$NH(CH_3)_2$-), 1.9 (6H, -CH_2C-), 1.46 (11H, -N^+-$CH_2(CH_2)_4CH_3$-), 0.98 (9H, -CH_2CCH_3-)

P(DMAEMA-co-Q_{12}DMAEMA)-b-POEGMA, 600 MHZ, D2O-d1, δ): 4.38 (2H, - $COOCH_2$ $CH_2N^+((CH_3)_2(CH_2))$-) 4.23 (2H, -($COOCH_2$ CH_2N-), 3.72 (36H, -$(CH_2CH_2O)_9CH_3$) 3.54 (2H, - $COOCH_2$ $CH_2N^+((CH_3)_2(CH_2))$-), -), 3.43 (3H, -$(CH_2CH_2O)_9CH_3$-), 3.38 (6H, - $COOCH_2$ $CH_2N^+((CH_3)_2(CH_2))$-), 2.84 (2H, - $COOCH_2$ $CH_2N^+((CH_3)_2(CH_2))$-) , 2.69 (2H, -($COOCH_2$ CH_2N-) , 2.41 (6H, -(6H, -$NH(CH_3)_2$-), 1.99 (6H, -CH_2C-), 1.47 (11H, -N^+-$CH_2(CH_2)_{10}CH_3$-), 0.97 (9H, -CH_2CCH_3-)

2. Additional Results

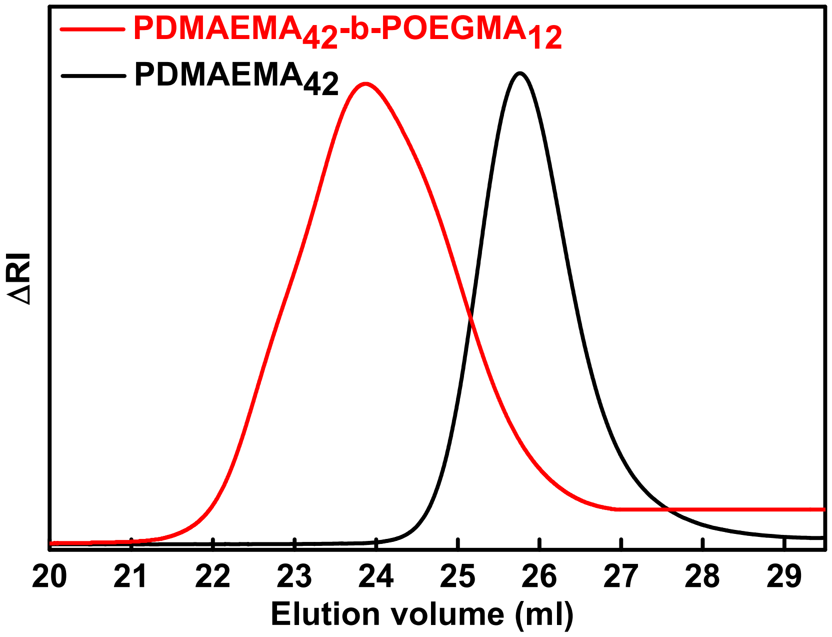

Figure S1. SEC chromatograms of $PDMAEMA_{42}$ (first block) and $PDMAEMA_{42}$-b-$POEGMA_{12}$ (final diblock copolymer).

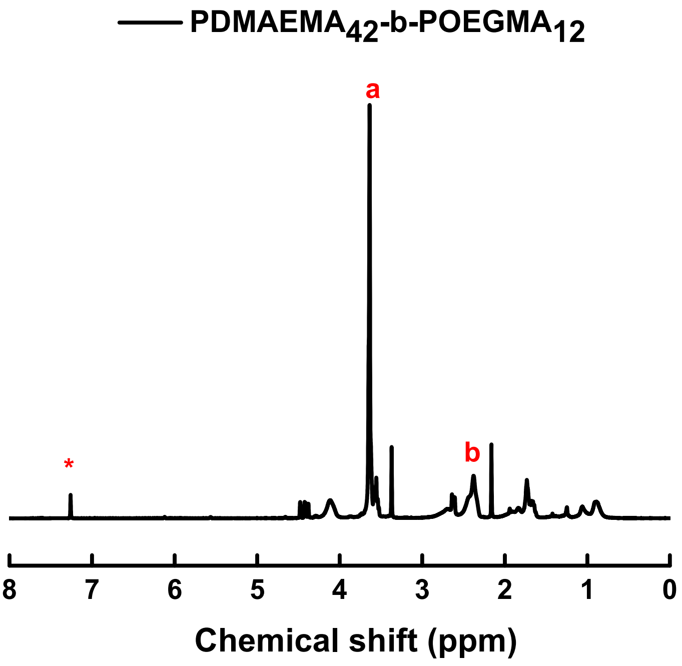

Figure S2. ^{1}H-NMR spectrum for $PDMAEMA_{44}$-b-$POEGMA_{12}$ diblock in $CDCl_3$. (The weight composition of the POEGMA block was determined by integrating the peak of the methylene proton groups at 3.62 ppm (peak a) and of the PDMAEMA block by integrating the peak of methyl proton groups at 2.36 ppm (peak b)).

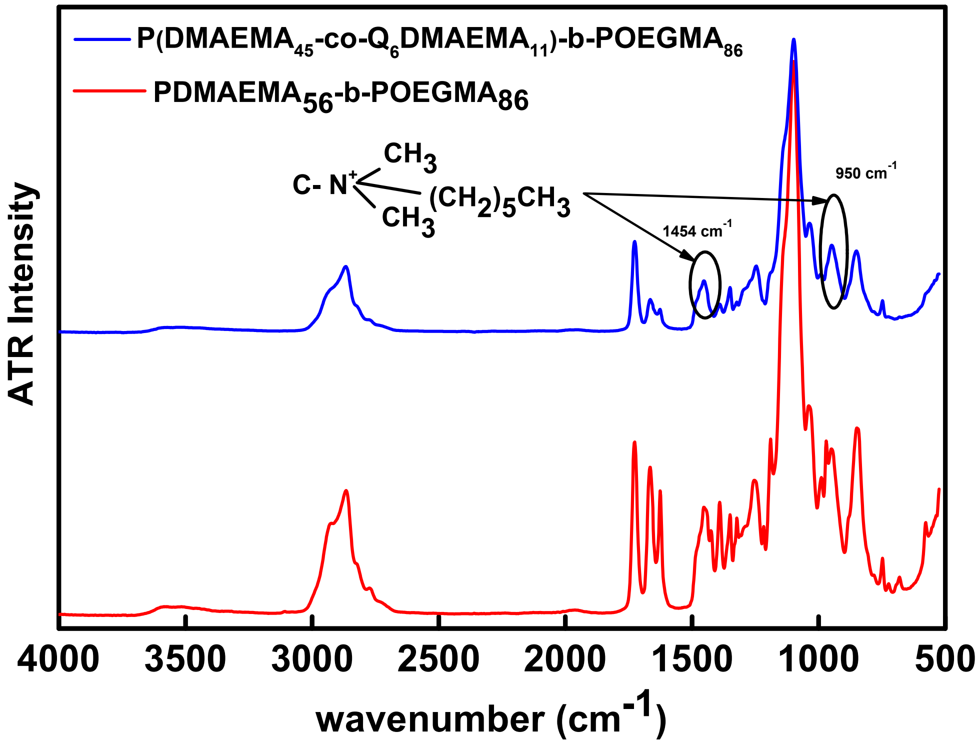

Figure S3. Comparative ATR-FTIR spectra of (a) PDMAEMA56-b-POEGMA86 precursor diblock copolymer and (b) P(DMAEMA45-b-Q6DMAEMA11)-b-POEGMA86 resulted random diblock copolymer .In the ATR-FTIR spectrum of the derivative, the presence of new absorption peaks at 1454 cm^{-1} and 950 cm^{-1} confirms the existence of quaternary amine groups.

Table S1. Scattering intensity and R_h results for the random diblock copolymers, obtained by the partial quaternization of the PDMAEMA56-b-POEGMA86 precursor

Direct dissolution in aqueous medium preparation protocol		
C = 1 x 10^{-3} g/mL, pH = 7, T = 25°C		
Sample	Intensity [a] (kc/s)	R_h [b] (nm)
P(DMAEMA45-co-Q6DMAEMA11)-b-POEGMA86 (1Q6)	25 ± 5	5.63 ±1
P(DMAEMA30-co-Q6DMAEMA26)-b-POEGMA86 (2Q6)	23 ± 5	5.27 ±1
P(DMAEMA44-co-Q12DMAEMA12)-b-POEGMA86 (1Q12)	28 ± 5	5.89 ±1
P(DMAEMA28-co-Q12DMAEMA28)-b-POEGMA86 (2Q12)	30 ± 5	5.42 ±1

[a] Determined by SLS, [b] Determined by DLS, at measuring angle 90°

pH-response of the Derivatives of the PDMAEMA42-b-POEGMA12 Copolymer Using the Organic Solvent Preparation Protocol

The preparation of aqueous solutions of the random diblock copolymers derived by the partial quaternization of the PDMAEMA42-b-POEGMA12, using the direct dissolution protocol in the aqueous medium, was not possible due to the high content of PDMAEMA block that resulted in a strong hydrophobic character as a large number of long hydrophobic alkyl chains were finally attached to the amino groups. Aqueous solutions of the

particular modified diblocks were prepared by the organic solvent preparation protocol. Based on the DLS measu rements on these solutions conducted at three different pHs, no significant changes in size and scattered intensity of the formed aggregates was monitored. The latter observation may be correlated to the morphology of the nanoaggregates formed as a result of the self-assembly at pH=7. The long hydrophobic chains probably hide the pH-sensitive amino groups of the DMAEMA segments and hydrogen ions do not have direct access to them or protonation/deprotonation of DMAEMA segments cannot overcome existing hydrophobic associations. Interesting results were obtained regarding the R_g/R_{h0} ratio of the aggregates in the solutions. The R_g/R_{h0} ratio of $3Q_6$ diblock aggregates is 1.1, a value that may indicate the formation of spherical vesicles [2]. This value is also observed in the case of $4Q_6$ diblock at all three different pH values, indicating that the formation of spherical vesicles is not affected by pH variations. The R_g/R_{h0} ratios of $4Q_{12}$ at pH 7 (=1.05), at pH 3 (=1) and pH 10 (=0.99) also suggest the formation of spherical vesicles. The scattering intensity and the hydrodynamic radius as a function of pH alterations are presented in Figures S4 and S5.

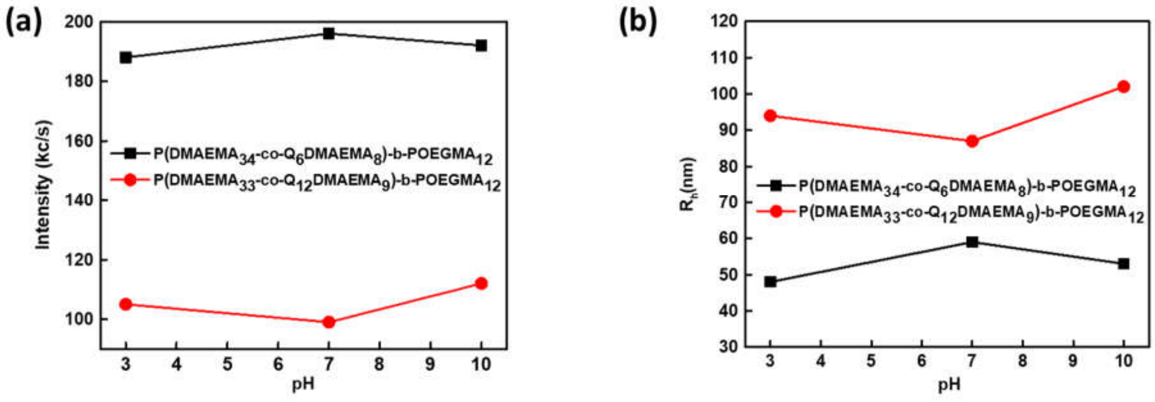

Figure S4. Dependence of (a) scattering intensity (I) and (b) hydrodynamic radius of $P(DMAEMA_{34}\text{-co-}Q_6DMAEMA_8)\text{-b-}POEGMA_{12}$ and $P(DMAEMA_{33}\text{-co-}Q_{12}DMAEMA_9)\text{-b-}POEGMA_{12}$ aqueous solutions on pH-variations (THF was used as the organic solvent to facilitate copolymer solubilization).

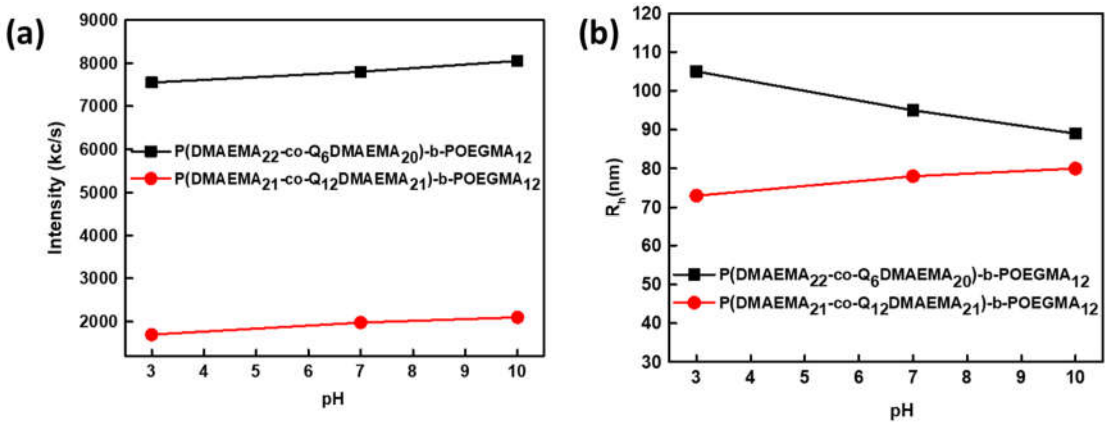

Figure S5. Dependence of (a) scattering intensity (I) and (b) hydrodynamic radius of $P(DMAEMA_{22}\text{-co-}Q_6DMAEMA_{20})\text{-b-}POEGMA_{12}$ and $P(DMAEMA_{21}\text{-co-}Q_{12}DMAEMA_{21})\text{-b-}POEGMA_{12}$ aqueous solutions on pH-variations (THF solubilization protocol)

Table S2. Scattering intensity and R_h results for the $1Q_6$ and $1Q_{12}$ diblocks at T=25 °C and T=45 °C

Organic solvent preparation protocol			
C = 1 x 10^{-3} g/mL, pH = 7			
Sample	T (°C)	Intensity [a] (kc/s)	R_h [b] (nm)
P($DMAEMA_{45}$-co-$Q6DMAEMA_{11}$)-b-$POEGMA_{86}$ ($1Q_6$)	25	633 ± 10	78 ± 2
	45	665 ± 10	82 ± 2
P($DMAEMA_{44}$-co-$Q12DMAEMA_{12}$)-b-$POEGMA_{86}$ ($1Q_{12}$)	25	805 ± 10	94 ± 2
	45	820 ± 10	97 ± 2

[a] Determined by SLS, [b] Determined by DLS, at measuring angle 90°

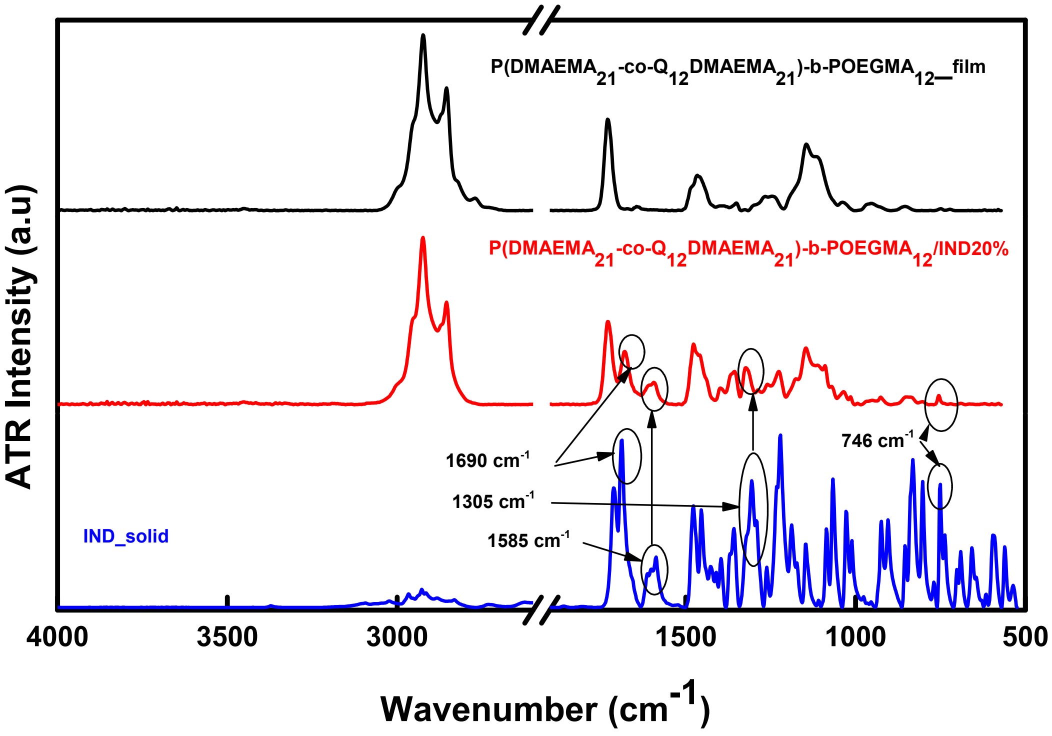

Figure S6. Comparative ATR-FTIR spectra of the mixed P($DMAEMA_{21}$-co-$Q_{12}DMAEMA_{21}$)-b-$POEGMA_{12}$/IND20% (black line), the P($DMAEMA_{21}$-co-$Q_{12}DMAEMA_{21}$)-b-$POEGMA_{12}$ empty vector (red line) and IND in the solid state (blue line). Note: The ATR-FTIR spectra of aqueous solutions of random diblock copolymer and mixed nanoassemblies of random diblock copolymer with IND emanated after the evaporation of water by using nitrogen, so that the water absorption peaks would not intervene with the characteristic absorption peaks that would reveal the interactions between IND and the random diblock copolymer. The correspondence of the absorption peaks to the functional groups of IND are analyzed in the main text.

References

1. Vuoriluoto, M.; Orelma, H.; Johansson, L.S.; Zhu, B.; Poutanen, M.; Walther, A.; Laine, J.; Rojas, O.J. Effect of Molecular Architecture of PDMAEMA-POEGMA Random and Block Copolymers on Their Adsorption on Regenerated and Anionic Nanocelluloses and Evidence of Interfacial Water Expulsion. *J. Phys. Chem. B* **2015**, *119*, 15275-15286. doi:10.1021/acs.jpcb.5b07628.
2. Ohno, S.; Ishihara, K.; Yusa, S. Formation of Polyion Complex (PIC) Micelles and Vesicles with Anionic pH-Responsive Unimer Micelles and Cationic Diblock Copolymers in Water. *Langmuir* **2016**, *32*, 3945-3953. doi:10.1021/acs.langmuir.6b00637.
